# Modelling and simulation of biased agonism dynamics at a G protein-coupled receptor

**DOI:** 10.1016/j.jtbi.2018.01.010

**Published:** 2018-04-07

**Authors:** L.J. Bridge, J. Mead, E. Frattini, I. Winfield, G. Ladds

**Affiliations:** a Department of Mathematics, Swansea University, Singleton Park, Swansea SA2 8PP, UK; b Department of Engineering Design and Mathematics, University of the West of England, Frenchay Campus, Bristol BS16 1QY, UK; c Department of Pharmacology, University of Cambridge, Tennis Court Road, Cambridge CB2 1PD, UK; d Division of Biomedical Sciences, Warwick Medical School, University of Warwick, Coventry CV4 7AL, UK

**Keywords:** Mathematical pharmacology, G protein-coupled receptors, Receptor theory, Biased signalling, Ordinary differential equations

## Abstract

Theoretical models of G protein-coupled receptor (GPCR) concentration-response relationships often assume an agonist producing a single functional response via a single active state of the receptor. These models have largely been analysed assuming steady-state conditions. There is now much experimental evidence to suggest that many GPCRs can exist in multiple receptor conformations and elicit numerous functional responses, with ligands having the potential to activate different signalling pathways to varying extents–a concept referred to as biased agonism, functional selectivity or pluri-dimensional efficacy. Moreover, recent experimental results indicate a clear possibility for time-dependent bias, whereby an agonist’s bias with respect to different pathways may vary dynamically. Efforts towards understanding the implications of temporal bias by characterising and quantifying ligand effects on multiple pathways will clearly be aided by extending current equilibrium binding and biased activation models to include G protein activation dynamics. Here, we present a new model of time-dependent biased agonism, based on ordinary differential equations for multiple cubic ternary complex activation models with G protein cycle dynamics. This model allows simulation and analysis of multi-pathway activation bias dynamics at a single receptor for the first time, at the level of active G protein (*α_GTP_*), towards the analysis of dynamic functional responses. The model is generally applicable to systems with *N^G^* G proteins and N* active receptor states. Numerical simulations for $N^{G}=N^{*}=2$ reveal new insights into the effects of system parameters (including cooperativities, and ligand and receptor concentrations) on bias dynamics, highlighting new phenomena including the dynamic inter-conversion of bias direction. Further, we fit this model to ‘wet’ experimental data for two competing G proteins (*G_i_* and *G_s_*) that become activated upon stimulation of the adenosine A_1_ receptor with adenosine derivative compounds. Finally, we show that our model can qualitatively describe the temporal dynamics of this competing G protein activation.

## Highlights

• A new ordinary differential equation model for biased agonism dynamics at a G protein-coupled receptor (GPCR) is presented.
• The model is general in the number of G proteins and active receptor.
• Numerical simulations reveal new phenomena in active G protein dynamics, including inter-conversion of the observed ligand effect (agonist to inverse agonist).
• The model recapitulates new experimental data for cells and ligands which are believed to exhibit biased agonism.

## 1 Introduction

Mathematical modelling and scientific computing are powerful tools for the analysis of cell signalling in pharmacology. “Analytical pharmacology”, which has its roots in classical receptor theory and largely focuses on equilibrium cell responses to drugs, provides a vital theoretical basis which underpins drug classification and prediction of drug mechanism of action (Kenakin and Christopoulos, 2011). Much of the analysis has centred on assumptions of a single ligand binding a monomeric G protein-coupled receptor (GPCR), activating a single active state and coupling a single G protein. Concepts like allosterism, inverse agonism, oligomerisation and “biased signalling” are now widely accepted and have enhanced receptor theory towards better understanding of drug-receptor interactions and informed drug discovery (Kenakin and Williams, 2014). GPCRs represent a target for perhaps up to half of all current drugs (Woodroffe et al., 2009), and as such, development of the theory for ligand-GPCR interactions and their consequences is key.

*Biased agonism* is now a widely accepted phenomenon whereby a ligand may activate multiple different pathways at the same receptor, via multiple active conformations (Kenakin, 2011, Onaran, Rajagopal, Costa, 2014, Rankovic, Brust, Bohn, 2016, Urban, Clarke, Von Zastrow, Nichols, Kobilka, Weinstein, Javitch, Roth, Christopoulos, Sexton, et al., 2007). Other terms for this phenomenon include *functional selectivity* and *pluri-dimensional efficacy*, while *receptor promiscuity* refers to the ability of a receptor to couple different G proteins with different affinities, via different active states. The possibility of multi-pathway activation may lead to a breakdown in the common classifications of ligands based on single active state theory (Kenakin, 2011), or errors in the interpretation of data using simple models (Tuček et al., 2002). Therefore, development of biased agonism theory has become an important field of pharmacological research.

**Fig. 1:**
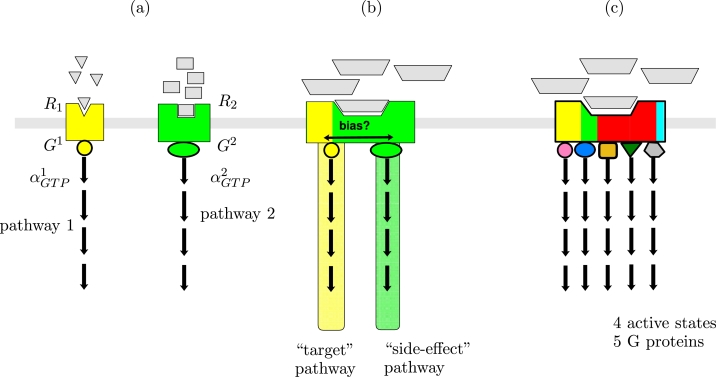
Pluri-dimensional efficacy and biased agonism at a GPCR. (a) A classical view of signalling–two different receptors, each bound and activated (to a single active conformation) by a specific ligand, and bound by a specific G protein. The activated G protein subunit *α_GTP_* signals to a downstream pathway specific to the G protein. (b) A two-active-state, two-G protein biased signalling schematic. The receptor has two active states, and the proportion of receptors in either active state, and the inactive state, may be affected (biased) by a single ligand. Two different G proteins, specific to the active conformations, couple to the receptors and signal to two pathways. (c) Pluri-dimensional efficacy - multi-active receptor with multiple G proteins, not necessarily each specific to a single receptor conformation. Here we have $N^{*}=4$ active states (represented by yellow, green, red and blue in the receptor block) and $N^{G}=5$ G proteins. (For interpretation of the references to colour in this figure legend, the reader is referred to the web version of this article.)

Biased signalling has implications for drug discovery, including the prospect of clinical selectivity and the potential of reduced side effects (Kenakin, 2015, Kenakin, Christopoulos, 2013, Stott, Hall, Holliday, 2015). A schematic of biased agonism is shown in Fig. 1, indicating possibility for a ligand to activate two (or more) G protein pathways at the same receptor, one of which may be a “target” therapeutic pathway, while the other may be an unwanted “side-effect” pathway (panel (b)). To understand, quantify and exploit the potential for biased agonism, theoretical models for such schematics are required.

A two-active-state model of ligand binding and receptor activation at equilibrium was presented in Leff et al. (1997). This equilibrium model addressed the limitations of single-active-state theory which could not recapitulate different pathway potency and efficacy patterns at the same receptor. It was found that theoretically, an agonist may enrich one active receptor state at the expense of another, and pathway-dependent efficacy was observed in simulations. For an intact system, however, pathway-dependent potency (with active receptor as the pathway readout) was not possible. G protein coupling and activation were not explicitly modelled, but their importance for future modelling was acknowledged. Later equilibrium models included the binding of G proteins (Ehlert, 2008, Scaramellini, Leff, 2002), which give further scope for pathway-dependent pharmacology. An alternative model for biased agonism is given in Roche et al. (2013), where downstream effects are modelled not explicitly via G protein binding, but by coupling the operational model of agonism (Black and Leff, 1983) to active receptor stimuli. This model does not include constitutive activity of the receptors. Further equilibrium modelling for promiscuous coupling of receptors to multiple G proteins has been presented in Kukkonen et al. (2001) and Tuček et al. (2002).

The direction and magnitude of a ligand’s bias towards one pathway over another has largely been quantified using equilibrium assumptions and empirical models such as the operational model (Gundry, Glenn, Alagesan, Rajagopal, 2017, Kenakin, 2014, Kenakin, Watson, Muniz-Medina, Christopoulos, Novick, 2012, Rajagopal, Ahn, Rominger, Gowen MacDonald, Lam, DeWire, Violin, Lefkowitz, 2011). A recent study (Herenbrink et al., 2016) has highlighted the role of “kinetic context” in approaching such calculations, whereby the apparent bias of a ligand towards any given pathway may vary over time. Interpretation of experimental readouts in terms of bias must therefore take into account the signalling dynamics and associated timescales of the measured pathway. Thus, dynamic models of GPCR biased signalling are proposed here to give new theoretical insights into the effects of biased agonists.

In Chen et al. (2003), an ordinary differential equation (ODE) model for the dynamics of biased signalling at GPCRs is presented. The steady-state behaviour of the model is analysed, with particular attention paid to the effect of G protein concentration, where the model output is active G protein. The dynamics in Chen et al. (2003) are not examined in detail, but extensive analysis of GPCR signalling dynamics has been presented elsewhere (Bridge, King, Hill, Owen, 2010, Woodroffe, Bridge, King, Chen, Hill, 2010, Woodroffe, Bridge, King, Hill, 2009), for mathematical models which also allow G proteins binding to inactive receptors, and constitutive receptor activity. In these models, the active G protein *α* subunit bound to guanosine triphosphate (*α_GTP_*) is taken as a model readout which is representative of downstream signalling pathway activity.

In this paper we develop a new mathematical model for the dynamics of biased agonism at GPCRs. The model allows an in-depth theoretical analysis of time-dependent biased agonism at a GPCR for the first time, and is novel in its generality and detail; any number of active receptor states and G proteins may be considered, receptor states need not be specific to particular G proteins, and the response is at the level of *α_GTP_*, downstream of active receptor and towards a dynamic functional response. In Section 2, we formulate a general ODE model for the dynamics of a receptor which can activate multiple G protein-mediated pathways. The general model has receptor with N* active conformations and *N^G^* G proteins available for coupling, but our focus computationally (driven by Leff et al., 1997) throughout is the case $N^{*}=N^{G}=2$. In Section 3, we present time course and concentration-response simulation results for our model, focusing on *α_GTP_* dynamics. In particular, we highlight that our model has the propensity for agonist-inverse agonist interconversion both with respect to time and constitutive activity. A numerical analysis of the effects of multiple cooperativity factors is performed. In Section 4, we propose an heuristic method for quantifying dynamic bias, by way of bias factors, and show how these bias factors relate to our model parameters. It is shown that the bias rank order for a bank of ligands may change dynamically. In Section 5, we show that our model simulations fit well to new experimental data where biased agonism at the adenosine *A*_1_ receptor is suspected. We conclude in Section 6 with a discussion of our main results, underlining our contribution to the biased signalling literature.

## 2 Model formulation

Here we formulate an ODE model for the dynamics of signalling for multi-active state GPCRs capable of binding multiple G proteins, in response to a single ligand binding. The model allows for a receptor which may have an inactive conformation *R*, or one of N* active receptor conformations R^*j^, for $j=1,\ldots ,N^{*}$. Also, a receptor may couple to one of *N^G^* G proteins *G^θ^*, for $\theta =1,\ldots ,N^{G}$. The model encompasses ligand binding, receptor activation, G protein binding and the G protein cycle, whereby the model output is activated G protein *α_GTP_*, which signals to second messengers, and is therefore taken as an indicator of pathway response, as in Woodroffe, Bridge, King, Chen, Hill, 2010, Woodroffe, Bridge, King, Hill, 2009 and Bridge et al. (2010).

### 2.1 A three-state (two active states) model

**Fig. 2:**
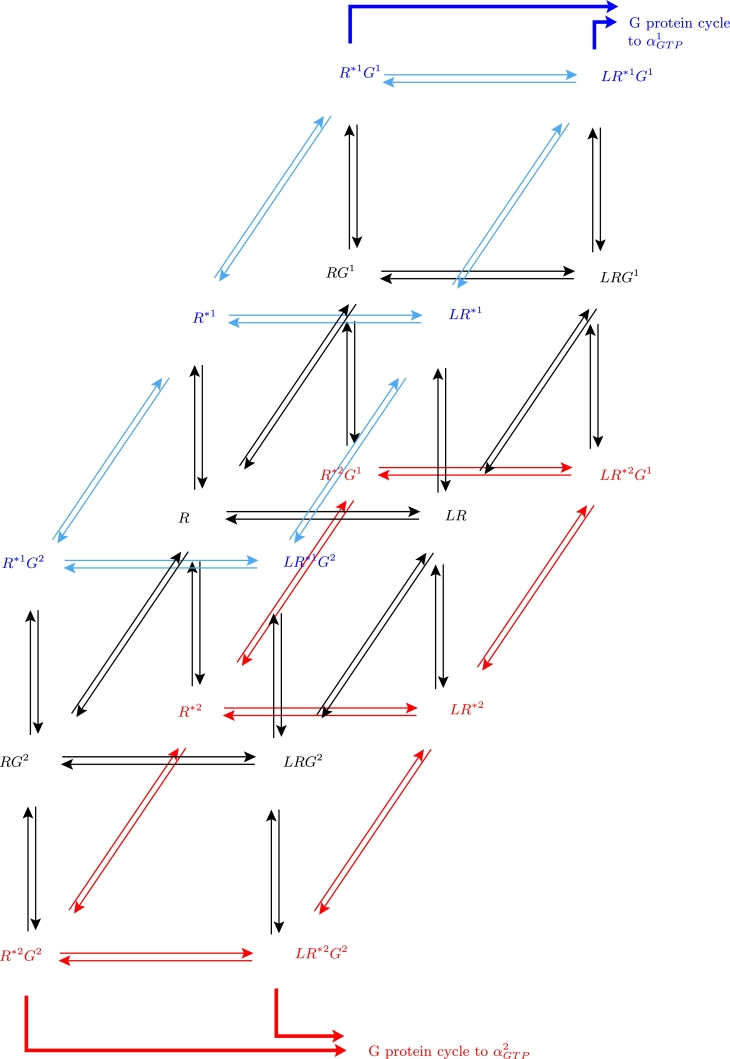
A multi-cubic ternary complex model schematic for biased signalling with two active receptor states (R^*1^, R^*2^) and two G proteins (*G*^1^, *G*^2^), giving 18 receptor species. Double arrows represent reversible binding and activation reactions between the receptor states. The four complexes R^*1^*G*^1^, LR^*1^*G*^1^, R^*2^*G*^2^ and LR^*2^*G*^2^ may dissociate, leading to the G protein cycle and increased active G protein signalling units $\alpha_{GTP}^{1}$ and $\alpha_{GTP}^{2}$.

While the model is formulated for general *N^G^* and N*, we largely focus throughout on the case $N^{*}=N^{G}=2$. A schematic for the transitions between 18 receptor states for this particular case is shown in Fig. 2. *R* denotes inactive receptor, while R^*j^ (j=1,2) denotes the *j*th active state. Any species label including *L* represents a complex including ligand-bound receptor, while any species including *G^θ^* (θ=1,2) is a complex including receptor coupled to the *θ*th G protein. Double arrows represent the reversible binding and activation reactions between receptor states. As described in previous GPCR signalling studies (eg. Bridge, King, Hill, Owen, 2010, Woodroffe, Bridge, King, Chen, Hill, 2010, Woodroffe, Bridge, King, Hill, 2009), a R*G^θ^ or LR*G^θ^ complex may dissociate and exchange *GDP* for *GTP* on the *α* subunit of the G protein, leading to the signalling *response* $\alpha_{GTP}^{\theta }$ and the G protein cycle.

### 2.2 The (*j, θ*) receptor/G protein block

**Fig. 3:**
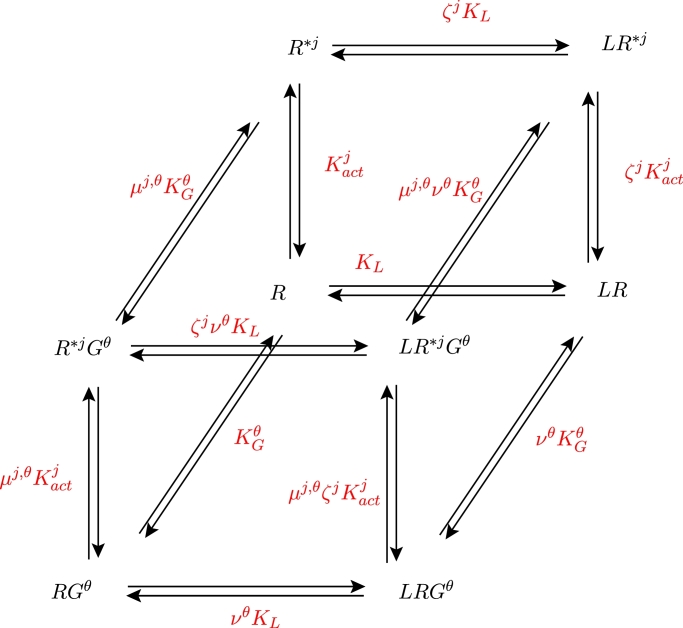
The (*j, θ*) receptor/G protein block of the multi-cubic ternary complex schematic, for ligand binding to, and activation of, receptor *j*, with coupling to G protein *θ*. Equilibrium rate constants *K*_•_ and cooperativity factors *μ, ν, ζ* are labelled on each reversible reaction.

In order to formulate the ODE model for the schematic shown in Fig. 2 (or, indeed, the general N*, *N^G^*-case), we consider the (*j, θ*) receptor/G protein block (where j=1,2 and θ=1,2 for Fig. 2). Each such block is seen to be a cubic ternary complex schema for activation of receptor from inactive state *R* to active state R^*j^, with coupling to G protein *G^θ^* (Woodroffe et al., 2009). In Fig. 3, the equilibrium rate constants *K*_•_ and cooperativity factors *μ, ν, ζ* are labelled on each reversible reaction. For the individual kinetic rate constants and factors, we use lower case *k*, and subscripts + and − to denote the forward and backward reactions respectively. The descriptions of the rate constants and cooperativity factors are given in Table 1. The G protein cycle and *α_GTP_* responses follow from dissociation of R*G^θ^ and LR*G^θ^ according to the following reactions (see Woodroffe et al., 2009):

(1a) $$\begin{array}{ccc} & & R^{*j}G^{\theta }\;\;\overset{k_{GTP+}^{j,\theta }}{⟶}\;\;R^{*j}+\alpha_{GTP}^{\theta }+\beta \gamma^{\theta },\\ & & LR^{*j}G^{\theta }\;\;\overset{\nu_{-}^{\theta }k_{GTP+}^{j,\theta }}{⟶}\;\;LR^{*j}+\alpha_{GTP}^{\theta }+\beta \gamma^{\theta }, \end{array}$$

(1b)
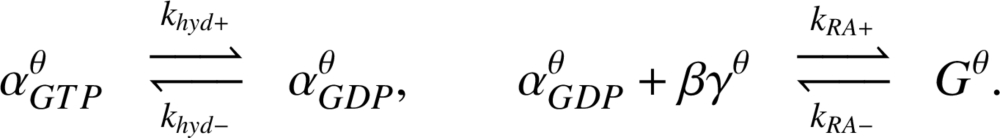

**Table 1:** Equilibrium rate constants and cooperativity factors for the (*j, θ*) block of the biased signalling schematic.

Label	Description of equilibrium constant
*K_L_*	Association of ligand *L* and receptor *R*.
$K_{G}^{\theta }$	Binding of G protein *G^θ^* to receptor *R*.
$K_{act}^{j}$	Activation of receptor *R* to give active state R^*j^.
*μ*^*j, θ*^	Preference of *G^θ^* for R^*j^ over *R*. Equally, the factor increase in propensity for *R* → R^*j^ activation when *R* is *G^θ^*-bound.
*ν^θ^*	Preference of *L* for *RG^θ^* over *R*. Equally, the preference of *G^θ^* for *LR* over *R*.
*ζ^j^*	Preference of *L* for R^*j^ over *R*. Equally, the factor increase in propensity for *R* → R^*j^ activation when *R* is *L*-bound.

#### 2.2.1 Governing equations

Suppose in general that a receptor has N* distinct active states, and that each receptor may couple one of *N^G^* distinct G proteins. Then applying mass action kinetics to our schematic and G protein cycle reactions gives a system of *n* nonlinear ODEs for the species concentrations, where

(2) $$n=3+2N^{*}+6N^{G}+2N^{*}N^{G}.$$

The first term here is given by species *L, R* and *LR*. The second term is given by active non-coupled receptor states R^*j^, LR^*j^ and the third term corresponds to G protein not coupled to active receptor (*G, RG, LRG*, $\alpha_{GTP}^{\theta },$ $\alpha_{GDP}^{\theta },$ *βγ^θ^*). Finally, the number of active receptor/G protein complexes, R^*j^*G^θ^* and LR^*j^*G^θ^*, is 2N*N^G^, since we consider $j=1,\ldots ,N^{*}$ and $\theta =1,\ldots ,N^{G}$. If ligand concentration is considered constant, then we will not have an ODE for [*L*] (so omitting Eq. (3b) below), and instead $n=2\left(1+N^{*}+3N^{G}+N^{*}N^{G}\right)$.

(3a) $$\begin{array}{cc} \frac{d[R]}{dt} & =k_{L-}\left[LR\right]-k_{L+}\left[L\right]\left[R\right]+\sum_{j=1}^{N^{*}}\left(k_{act-}^{j}\left[R^{*j}\right]-k_{act+}^{j}\left[R\right]\right)\\ & \;+\sum_{\theta =1}^{N^{G}}\left(k_{G-}^{\theta }\left[RG^{\theta }\right]-k_{G+}^{\theta }\left[R\right]\left[G^{\theta }\right]\right), \end{array}$$

(3b) $$\begin{array}{cc} \frac{d[L]}{dt} & =k_{L-}\left[LR\right]-k_{L+}\left[L\right]\left[R\right]+\sum_{j=1}^{N^{*}}\left(\zeta_{-}^{j}k_{L-}\left[LR^{*j}\right]-\zeta_{+}^{j}k_{L+}\left[L\right]\left[R^{*j}\right]\right)\\ & \;\;+\sum_{\theta =1}^{N^{G}}\left(\nu_{-}^{\theta }k_{L-}\left[LRG^{\theta }\right]-\nu_{+}^{\theta }k_{L+}\left[L\right]\left[RG^{\theta }\right]\right)\\ & \;\;+\sum_{\theta =1}^{N^{G}}\sum_{j=1}^{N^{*}}\left(\zeta_{-}^{j}\nu_{-}^{\theta }k_{L-}\left[LR^{*j}G^{\theta }\right]-\zeta_{+}^{j}\nu_{+}^{\theta }k_{L+}\left[L\right]\left[R^{*j}G^{\theta }\right]\right), \end{array}$$

(3c) $$\begin{array}{cc} \frac{d[LR]}{dt} & =k_{L+}\left[L\right]\left[R\right]-k_{L-}\left[LR\right]+\sum_{j=1}^{N^{*}}\left(\zeta_{-}^{j}k_{act-}^{j}\left[LR^{*j}\right]-\zeta_{+}^{j}k_{act+}^{j}\left[LR\right]\right)\\ & \;\;+\sum_{\theta =1}^{N^{G}}\left(\nu_{-}^{\theta }k_{G-}^{\theta }\left[LRG^{\theta }\right]-\nu_{+}^{\theta }k_{G+}^{\theta }\left[LR\right]\left[G^{\theta }\right]\right), \end{array}$$

(3d) $$\begin{array}{cc} \frac{d[R^{*j}]}{dt} & =k_{act+}^{j}\left[R\right]-k_{act-}^{j}\left[R^{*j}\right]+\zeta_{-}^{j}k_{L-}\left[LR^{*j}\right]-\zeta_{+}^{j}k_{L+}\left[L\right]\left[R^{*j}\right]\\ & \;+\sum_{\theta =1}^{N^{G}}\left(\mu_{-}^{j,\theta }k_{G-}^{\theta }\left[R^{*j}G^{\theta }\right]-\mu_{+}^{j,\theta }k_{G+}^{\theta }\left[R^{*j}\right]\left[G^{\theta }\right]\right)\\ & \;+\sum_{\theta =1}^{N^{G}}\left(k_{GTP+}^{j,\theta }\left[R^{*j}G^{\theta }\right]\right),\;\text{for}\;j=1,\ldots ,N^{*} \end{array}$$

(3e) $$\begin{array}{cc} \frac{d[LR^{*j}]}{dt} & =\zeta_{+}^{j}k_{act+}^{j}\left[LR\right]-\zeta_{-}^{j}k_{act-}^{j}\left[LR^{*j}\right]+\zeta_{+}^{j}k_{L+}\left[L\right]\left[R^{*j}\right]-\zeta_{-}^{j}k_{L-}\left[LR^{*j}\right]\\ & \;+\sum_{\theta =1}^{N^{G}}\left(\mu_{-}^{j,\theta }\nu_{-}^{\theta }k_{G-}^{\theta }\left[LR^{*j}G^{\theta }\right]-\mu_{+}^{j,\theta }\nu_{+}^{\theta }k_{G+}^{\theta }\left[LR^{*j}\right]\left[G^{\theta }\right]\right)\\ & \;+\sum_{\theta =1}^{N^{G}}\left(\nu_{-}^{\theta }k_{GTP+}^{j,\theta }\left[LR^{*j}G^{\theta }\right]\right),\;\text{for}\;j=1,\ldots ,N^{*} \end{array}$$

(3f) $$\begin{array}{cc} \frac{d[RG^{\theta }]}{dt} & =k_{G+}^{\theta }\left[R\right]\left[G^{\theta }\right]-k_{G-}^{\theta }\left[RG^{\theta }\right]+\nu_{-}^{\theta }k_{L-}\left[LRG^{\theta }\right]-\nu_{+}^{\theta }k_{L+}\left[L\right]\left[RG^{\theta }\right]\\ & \;\;+\sum_{j=1}^{N^{*}}\left(\mu_{-}^{j,\theta }k_{act-}^{j}\left[R^{*j}G^{\theta }\right]-\mu_{+}^{j,\theta }k_{act+}^{j}\left[RG^{\theta }\right]\right),\\ \text{for}\;\theta & =1,\ldots ,N^{G}, \end{array}$$

(3g) $$\begin{array}{cc} \frac{d[LRG^{\theta }]}{dt} & =\nu_{+}^{\theta }k_{G+}^{\theta }\left[LR\right]\left[G^{\theta }\right]-\nu_{-}^{\theta }k_{G-}^{\theta }\left[LRG^{\theta }\right]\\ & \;+\nu_{+}^{\theta }k_{L+}\left[L\right]\left[RG^{\theta }\right]-\nu_{-}^{\theta }k_{L-}\left[LRG^{\theta }\right]\\ & \;+\sum_{j=1}^{N^{*}}\left(\mu_{-}^{j,\theta }\zeta_{-}^{j}k_{act-}^{j}\left[LR^{*j}G^{\theta }\right]-\mu_{+}^{j,\theta }\zeta_{+}^{j}k_{act+}^{j}\left[LRG^{\theta }\right]\right),\\ \text{for}\;\theta & =1,\ldots ,N^{G}, \end{array}$$

(3h) $$\begin{array}{cc} \frac{d[R^{*j}G^{\theta }]}{dt} & =\mu_{+}^{j,\theta }k_{G+}^{\theta }\left[R^{*j}\right]\left[G^{\theta }\right]-\mu_{-}^{j,\theta }k_{G-}^{\theta }\left[R^{*j}G^{\theta }\right]\\ & \;+\zeta_{-}^{j}\nu_{-}^{\theta }k_{L-}\left[LR^{*j}G^{\theta }\right]-\zeta_{+}^{j}\nu_{+}^{\theta }k_{L+}\left[L\right]\left[R^{*j}G^{\theta }\right]\\ & \;+\mu_{+}^{j,\theta }k_{act+}^{j}\left[RG^{\theta }\right]-\mu_{-}^{j,\theta }k_{act-}^{j}\left[R^{*j}G^{\theta }\right]\\ & \;\;-k_{GTP+}^{j,\theta }\left[R^{*j}G^{\theta }\right]\\ \text{for}\;j & =1,\ldots ,N^{*}\;\text{and}\;\theta =1,\ldots ,N^{G}, \end{array}$$

(3i) $$\begin{array}{cc} \frac{d[LR^{*j}G^{\theta }]}{dt} & =\mu_{+}^{j,\theta }\nu_{+}^{\theta }k_{G+}^{\theta }\left[LR^{*j}\right]\left[G^{\theta }\right]-\mu_{-}^{j,\theta }\nu_{-}^{\theta }k_{G-}^{\theta }\left[LR^{*j}G^{\theta }\right]\\ & \;+\zeta_{+}^{j}\nu_{+}^{\theta }k_{L+}\left[L\right]\left[R^{*j}G^{\theta }\right]-\zeta_{-}^{j}\nu_{-}^{\theta }k_{L-}\left[LR^{*j}G^{\theta }\right]\\ & \;+\mu_{+}^{j,\theta }\zeta_{+}^{j}k_{act+}^{j}\left[LRG^{\theta }\right]-\mu_{-}^{j,\theta }\zeta_{-}^{j}k_{act-}^{j}\left[LR^{*j}G^{\theta }\right]\\ & \;-\nu_{-}^{\theta }k_{GTP+}^{j,\theta }\left[LR^{*j}G^{\theta }\right]\\ \text{for}\;j & =1,\ldots ,N^{*}\;\text{and}\;\theta =1,\ldots ,N^{G}, \end{array}$$

(3j) $$\begin{array}{cc} \frac{d[G^{\theta }]}{dt} & =k_{G-}^{\theta }\left[RG^{\theta }\right]-k_{G+}^{\theta }\left[R\right]\left[G^{\theta }\right]\;+\;\nu_{-}^{\theta }k_{G-}^{\theta }\left[LRG^{\theta }\right]-\nu_{+}^{\theta }k_{G+}^{\theta }\left[LR\right]\left[G^{\theta }\right]\\ & \;+k_{GRA+}^{\theta }\left[\alpha_{GDP}^{\theta }\right]\left[\beta \gamma^{\theta }\right]-k_{GRA-}^{\theta }\left[G^{\theta }\right]\\ & \;+\sum_{j=1}^{N^{*}}\left(\mu_{-}^{j,\theta }k_{G-}^{\theta }\left[R^{*j}G^{\theta }\right]-\mu_{+}^{j,\theta }k_{G+}^{\theta }\left[R^{*j}\right]\left[G^{\theta }\right]\right)\\ & \;+\sum_{j=1}^{N^{*}}\left(\mu_{-}^{j,\theta }\nu_{-}^{\theta }k_{G-}^{\theta }\left[LR^{*j}G^{\theta }\right]-\mu_{+}^{j,\theta }\nu_{+}^{\theta }k_{G+}^{\theta }\left[LR^{*j}\right]\left[G^{\theta }\right]\right),\\ \text{for}\;\theta & =1,\ldots ,N^{G}, \end{array}$$

(3k) $$\begin{array}{cc} \frac{d[\alpha_{GDP}^{\theta }]}{dt} & =k_{hyd+}^{\theta }\left[\alpha_{GTP}^{\theta }\right]-k_{hyd-}^{\theta }\left[\alpha_{GDP}^{\theta }\right]+k_{GRA-}^{\theta }\left[G^{\theta }\right]\\ & \;-k_{GRA+}^{\theta }\left[\alpha_{GDP}^{\theta }\right]\left[\beta \gamma^{\theta }\right],\\ \text{for}\;\theta & =1,\ldots ,N^{G}, \end{array}$$

(3l) $$\begin{array}{cc} \frac{d[\beta \gamma^{\theta }]}{dt} & =k_{GRA-}^{\theta }\left[G^{\theta }\right]-k_{GRA+}^{\theta }\left[\alpha_{GDP}^{\theta }\right]\left[\beta \gamma^{\theta }\right]\\ & \;+\sum_{j=1}^{N^{*}}\left(k_{GTP+}^{j,\theta }\left[R^{*j}G^{\theta }\right]+\nu_{-}^{\theta }k_{GTP+}^{j,\theta }\left[LR^{*j}G^{\theta }\right]\right),\\ \text{for}\;\theta & =1,\ldots ,N^{G}, \end{array}$$

(3m) $$\begin{array}{cc} \frac{d[\alpha_{GTP}^{\theta }]}{dt} & =k_{hyd-}^{\theta }\left[\alpha_{GDP}^{\theta }\right]-k_{hyd+}^{\theta }\left[\alpha_{GTP}^{\theta }\right]\\ & \;+\sum_{j=1}^{N^{*}}\left(k_{GTP+}^{j,\theta }\left[R^{*j}G^{\theta }\right]+\nu_{-}^{\theta }k_{GTP+}^{j,\theta }\left[LR^{*j}G^{\theta }\right]\right),\\ \text{for}\;\theta & =1,\ldots ,N^{G}. \end{array}$$

For the model “outputs”, or downstream responses of the system to an input ligand concentration, we take the concentrations $\left[\alpha_{GTP}^{\theta }\right]$ for $\theta =1,\ldots ,N^{G},$ as we consider these as indicators of downstream activity in signalling pathways as in Bridge (2009) and Bridge et al. (2010). For our computational results, we will consider the case with two G proteins and two active receptor states, such that $N^{*}=N^{G}=2,$ and our model has 18 receptor states and 8 non-receptor-bound G protein species ($2\times (G+\alpha_{GTP}+\alpha_{GDP}+\beta \gamma )$). Taking ligand concentration constant (as in previous studies), the system (3) in this case consists of 26 ODEs.

Initial conditions for our simulations have ${\left[R\right]}_{t=0}=R_{tot}$ (the total receptor concentration), ${\left[G^{\theta }\right]}_{t=0}=G_{tot}^{\theta }$ (the total concentration for each G protein), and all other species zero at t=0.

## 3 Simulation results

Here we present numerical results (for $\alpha_{GTP}^{\theta }$ concentrations) which illustrate the variety of dynamic behaviour which is possible for a system of two active states and two-G proteins. These results are intended to demonstrate potential dynamics rather than provide exhaustive or accurate predictions for any particular receptors or ligands. For all simulations, we first compute the system with [L]=0 for a long time (10^8^ s) to allow the system to come to a steady-state equilibrium before the addition of ligand, and all parameters except those explicitly stated are maintained at the values in Table A.1.

### 3.1 Time courses

We consider time courses for the *two α_GTP_* responses of interest. The signifcance of these computations is that two responses $\left(\alpha_{GTP}^{1},\alpha_{GTP}^{2}\right)$ are generated from a single ligand at a single receptor, whereas previous GPCR dynamic models (eg. Bridge et al., 2010) have considered a single *α_GTP_* response from each receptor. Further, the dynamic responses for the new model do not necessarily follow the previously reported behaviour of ligands classed as agonists, antagonists or inverse agonists for a single active state. An agonist is a ligand which encourages receptor activation, an antagonist is neutral in its action, and an inverse agonist discourages receptor activation. Within our model, therefore, an agonist for active state R^*j^ has *ζ^j^* > 1, an antagonist has $\zeta^{j}=1,$ and an inverse agonist has *ζ^j^* < 1.

#### 3.1.1 Ligand is an agonist for both pathways

**Fig. 4:**
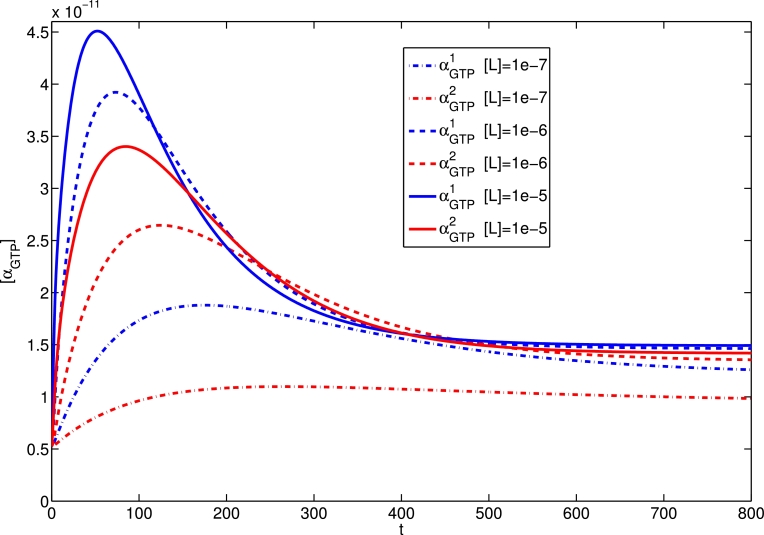
The $\alpha_{GTP}^{\theta }$ response (M) against time (in seconds) of two competing pathways with $\zeta_{+}^{1}=1000$ and $\zeta_{+}^{2}=200$ after the addition of $\left[L\right]=10^{-7},\;10^{-6}\;\text{and}\;10^{-5}$ M.

By varying the values of *ζ*^1^ and *ζ*^2^ , the preference of the ligand for a receptor in the active states 1 and 2 over the inactive receptor state, we vary the efficacy with respect to the G protein pathways 1 and 2 respectively. In Fig. 4, we show time courses of the responses to a ligand which is an equilibrium agonist for both pathways, for a range of concentrations. Three different ligand concentrations are used, and the $\alpha_{GTP}^{\theta }$ responses for θ=1,2 are shown. The higher efficacy with respect R^*1^ gives an increased response, and we note the peak-plateau dynamics. With increased ligand concentration, we see a higher $\alpha_{GTP}^{\theta }$ response for both pathways, both at peak and plateau (end-point). Further, the peak timing is reduced with increased ligand concentration, in keeping with previous single active state studies (Bridge, 2009, Woodroffe, Bridge, King, Hill, 2009).

#### 3.1.2 Ligand is agonist for one pathway and antagonist for the other

**Fig. 5:**
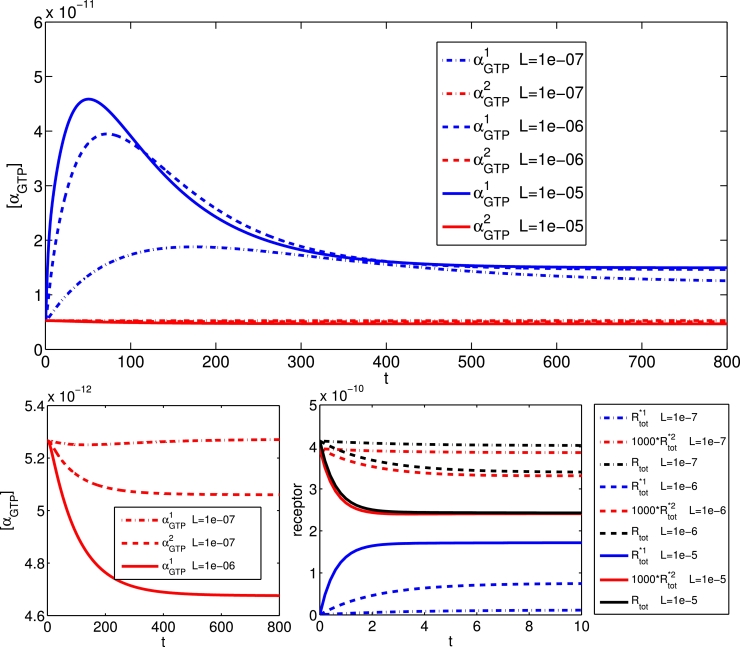
The $\alpha_{GTP}^{\theta }$ response (M) against time (in seconds) for two pathways with $\zeta_{+}^{1}=1000$ and $\zeta_{+}^{2}=1$ after the addition of $\left[L\right]=10^{-7},\;10^{-6},\;10^{-5}$ M so that the ligand is an agonist for pathway 1 and an antagonist for pathway 2. Receptor concentrations are given in the bottom panel. Here, $\zeta_{+}^{1}=1$.

Neutral antagonists may be used as competitive ligands to endogenous agonists. Mathematical modelling of agonist-antagonist competition at a single active state GPCR has been considered in Bridge et al. (2010). Within our two-active state model, we may simulate the dynamics of a system for which a given ligand is an agonist for one pathway but an antagonist for the other. In Fig. 5, we show *α_GTP_* and receptor time courses for this scenario, for a ligand which is an (equilibrium) agonist for pathway 1 ($\zeta_{+}^{1}=1000,$ $\zeta_{-}^{1}=1$) and an (equilibrium) antagonist for pathway 2 ($\zeta_{+}^{2}=1,$ $\zeta_{-}^{2}=1$), over a range of ligand concentrations. We note the peak-plateau $\alpha_{GTP}^{1}$ dynamics, and the nearly neutral effect on $\alpha_{GTP}^{2}$ dynamics. However, closer inspection of $\left[\alpha_{GTP}^{2}\right]$ reveals that the ligand in fact has an inverse agonist effect on pathway 2. Since the ligand is an agonist for pathway 1, its effect on overall receptor activation is an increase in pathway 1 active states, given by

(4) $$R_{tot}^{*1}=\left[R^{*1}\right]+\left[LR^{*1}\right]+\left[R^{*1}G^{1}\right]+\left[LR^{*1}G^{1}\right],$$

and a corresponding decrease in pathway 2 active states and free inactive receptor states, given, respectively, by

(5) $$R_{tot}^{*2}=\left[R^{*2}\right]+\left[LR^{*2}\right]+\left[R^{*2}G^{2}\right]+\left[LR^{*2}G^{2}\right],$$

and

(6) $$R_{tot}^{\text{inactive}}=\left[R\right]+\left[LR\right]+\left[RG^{1}\right]+\left[LRG^{1}\right]+\left[RG^{2}\right]+\left[LRG^{2}\right].$$

Upon ligand addition, there are, therefore, fewer receptors available to activate pathway 2, giving a decrease in $[\alpha_{GTP}^{2}],$ and the inverse agonist effect of the “antagonist”. For $\left[L\right]=10^{-7}$ M, we also see the undershoot *α_GTP_* response previously reported for inverse agonists (Bridge, 2009). We note that a true neutral antagonist effect with constant $\left[\alpha_{GTP}^{2}\right]$ would be seen if we considered pathway 2 as an “isolated pathway” (see Leff et al., 1997) by setting $k_{act+}^{1}=0$.

#### 3.1.3 Ligand is agonist for one pathway and inverse agonist for the other

**Fig. 6:**
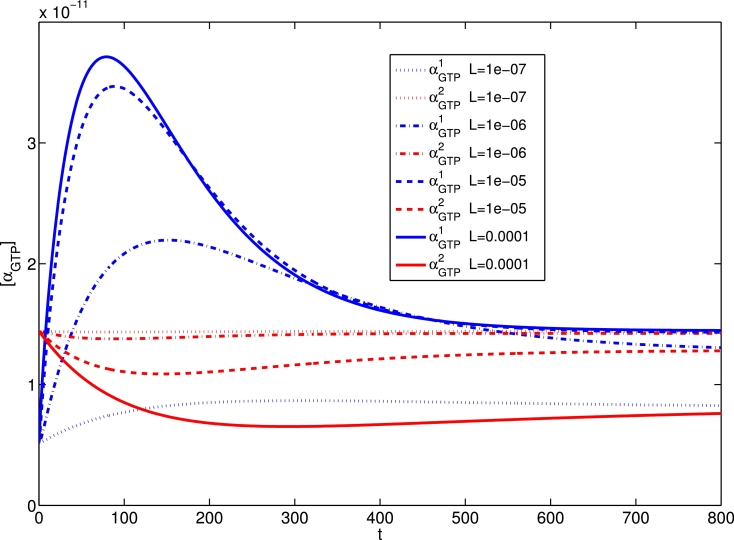
The $\alpha_{GTP}^{\theta }$ response (M) against time (s) after the addition of a range of ligand concentrations, where the ligand is an agonist for pathway 1 and an inverse agonist for pathway 2. Here, $\zeta_{+}^{1}=100,\;\zeta_{+}^{2}=0.01,\;k_{act-}^{2}=10$.

Having seen apparent inverse agonist activity in the biased system for a ligand which would neutrally antagonise an isolated pathway, we now turn attention to a ligand which is a true inverse agonist for one pathway in the biased system, and an agonist for the other. In Fig. 6, we show *α_GTP_* time courses for this scenario, for a ligand which is an (equilibrium) agonist for pathway 1 ($\zeta_{+}^{1}=100,$ $\zeta_{-}^{1}=1$) and an (equilibrium) inverse agonist for pathway 2 ($\zeta_{+}^{2}=0.01,$ $\zeta_{-}^{2}=1$), over a range of ligand concentrations. These simulations are for a system with increased R^*2^ constitutive activity, to represent conditions under which inverse agonism may be detectable. We note the peak-plateau $\alpha_{GTP}^{1}$ dynamics, and drop-off in $\alpha_{GTP}^{2}$ level. Further, we observe undershoot dynamics in the inversely agonised pathway, which may be seen in a single-active state system (Bridge, 2009). An interesting feature here is that while increasing ligand concentration decreases $\alpha_{GTP}^{1}$ peak time as before, this is accompanied by an increase in $\alpha_{GTP}^{2}$ trough time.

#### 3.1.4 Time course surfaces

**Fig. 7:**
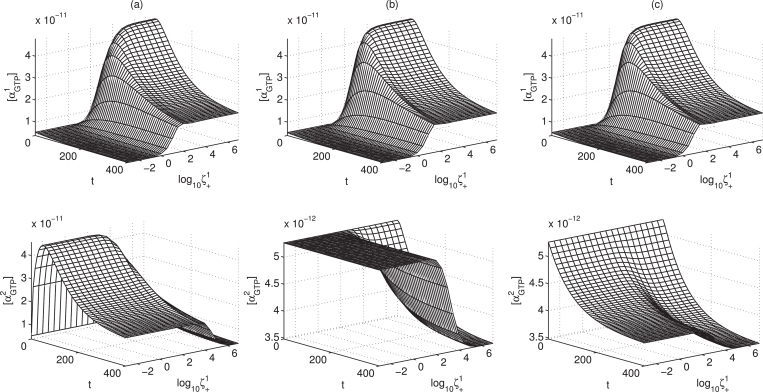
Time course surfaces for $\alpha_{GTP}^{\theta }$ response (M) dynamically changing for system with varying agonist efficacy parameter $\zeta_{+}^{1},$ acting under a ligand concentration of $10^{-5}$ M. Column (a): $\zeta_{+}^{2}=1000$; column (b): $\zeta_{+}^{2}=1$; column (c): $\zeta_{+}^{2}=0.001$.

In order to summarise the effect that efficacy parameter *ζ* has on a system, in Fig. 7 we show time course surfaces for $\left[L\right]=10^{-5}$ M, where we vary $\zeta_{+}^{1}$ over a spectrum of efficacy ranging from strong inverse agonist to strong agonist, while keeping all other parameters fixed. We clearly see that the stronger *L* is an agonist for pathway 1, the lesser its effect on pathway 2. When *L* is an agonist for both pathways, the peak-plateau dynamic response is clear for both $\alpha_{GTP}^{1}$ and $\alpha_{GTP}^{2},$ but increasing agonist strength for pathway 1, the pathway 2 response drops off. Similarly, for a pathway 2 antagonist, the $\alpha_{GTP}^{2}$ dynamic response varies from apparent antagonism to inverse agonism with increasing $\zeta_{+}^{1}$. Also, for a pathway 2 inverse agonist, the magnitude of $\alpha_{GTP}^{2}$ inverse agonism increases with $\zeta_{+}^{1}$.

#### 3.1.5 Observed agonist effect is system-dependent - constitutive activity and inter-conversion

**Fig. 8:**
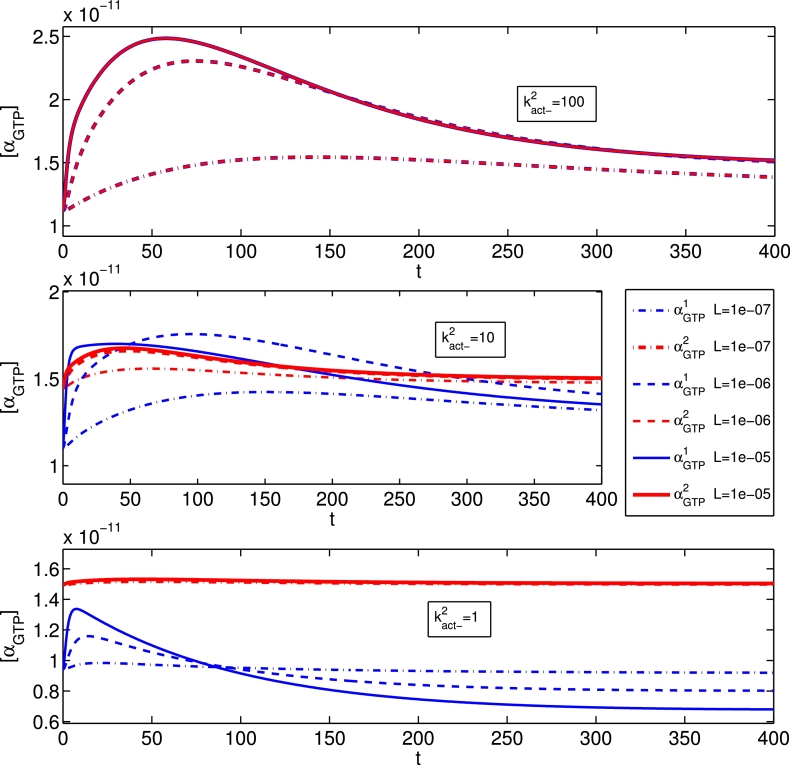
Time courses for $\alpha_{GTP}^{\theta }$ response (M) dynamically changing for systems with differing constitutive receptor activation level in pathway 2, varying $k_{act-}^{2}$. Here, $\zeta_{+}^{1}=\zeta_{+}^{2}=100,\;k_{act-}^{1}=100,\;k_{act-}^{2}=100$.

A feature of the equilibrium three-state model in Leff et al. (1997) is that a ligand’s effect on a pathway can change qualitatively from agonist to inverse agonist, depending on the system-specific level of constitutive activity in that pathway. This so-called “inter-conversion” of ligand effect is demonstrated at steady-state in Leff et al. (1997), with respect to active receptor states. In Fig. 8, we show the effects of increasing the constitutive activity in pathway 2 by decreasing $k_{act-}^{2}$. With low constitutive activity ($k_{act-}^{2}=100$), the time courses for $\alpha_{GTP}^{1,2}$ are indistinguishable. As pathway 2 constitutive activity is increased, it is clear that pathway 2 basal *α_GTP_* increases at the expense of pathway 1 basal *α_GTP_*, similarly to the active receptor trend in Leff et al. (1997) and Scaramellini and Leff (1998). The agonist effect on $\alpha_{GTP}^{2}$ becomes less pronounced with decreased $k_{act-}^{2},$ as the G protein response is largely effected via the basal activity, but the ligand remains a pathway 2 agonist. In contrast, with high activation of pathway 2 ($k_{act-}^{2}=1$), the long-time $\alpha_{GTP}^{1}$ response decreases with “agonist” concentration, so that the ligand is now having an apparent inverse agonist effect, despite its isolated pathway classification as an agonist. It is also worth noting that for ($k_{act-}^{2}=10$), we observe non-monotonicity in the peak and plateau $\alpha_{GTP}^{1}$ as functions of [*L*]. Thus non-monotonic concentration-response curves may result from multi-active state receptors with varying constitutive activity levels.

#### 3.1.6 Observed agonist effect may be time-dependent: G protein cycle and dynamic inter-conversion

With our new model, we are able to examine the $\alpha_{GTP}^{\theta }$ dynamics under variation of constitutive receptor activation. In the final plot of Fig. 8, we see the phenomenon of *dynamic inter-conversion* between agonist and inverse agonist action. The ligand is an agonist for both pathways under equilibrium classification, but after initially displaying a typical agonist response, $\alpha_{GTP}^{1}$ eventually drops below basal levels in an apparent inverse agonist response. The dynamic peak response to agonism occurs as in previous simulations (Woodroffe et al., 2009). The below basal long-time level is a result of G protein cycle dynamics on active receptor equilibration. As $\alpha_{GTP}^{1}$ is inactivated and *G*^1^ reassociates, any new free receptors resulting from *LRG* complex dissociation are pulled towards a pathway 2 dominant equilibrium, and the receptor pool for *G*^1^ activation decreases below basal level.

### 3.2 Concentration-response relationships

#### 3.2.1 Peak and plateau responses with varying ligand activation efficacy and constitutive receptor activity

**Fig. 9:**
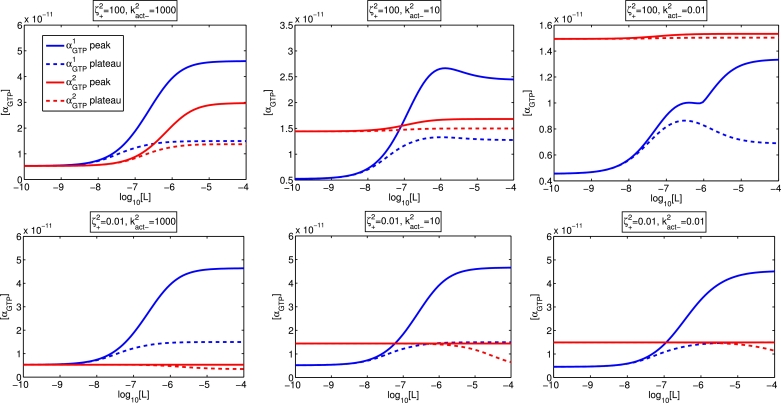
Concentration-response curves ($\alpha_{GTP}^{\theta }$ concentration against ligand concentration) where the ligand is an agonist for both pathways ($\zeta_{+}^{1}=1000,$$\zeta_{-}^{1}=1,$$\zeta_{+}^{2}=100,$$\zeta_{-}^{2}=1,$ top row) and agonist for pathway 1 but an inverse agonist for pathway 2 ($\zeta_{+}^{1}=1000,$$\zeta_{-}^{1}=1,$$\zeta_{+}^{2}=0.01,$$\zeta_{-}^{2}=1,$ bottom row). Constitutive activity for pathway 2 is low ($k_{act-}^{2}=1000,$ left column), medium ($k_{act-}^{2}=10,$ middle column), and high ($k_{act-}^{2}=1,$ left column). Here, $\zeta_{+}^{2}=100$.

The ligand concentration-dependent features which summarise the *α_GTP_* equilibrium and dynamic behaviour may be summarised using conventional concentration-response curves. In Fig. 9, we show concentration response curves for both pathways, where the measured responses are the peak and plateau *α_GTP_* levels. The non-monotonicity first noted in Section 3.1.5 is clearly a possibility. For a ligand which agonises both pathways, with high constitutive activity in one pathway, the plateau response in the other pathway is non-monotonic. The peak concentration-response curve is yet more complex; it is also non-monotonic, with a biphasic structure. We remark that biased agonism together with constitutive activity in our new model for *α_GTP_* response is a mechanism by which non-monotonic concentration-response relationships can occur. Such behaviour cannot be observed for three-state models (Leff, Scaramellini, Law, McKechnie, 1997, Scaramellini, Leff, 1998) where the “readout” is a particular active receptor fraction.

**Fig. 10:**
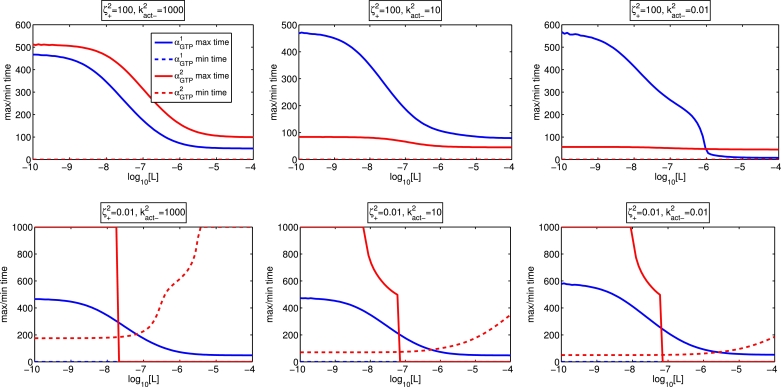
Concentration-response curves ($\alpha_{GTP}^{\theta }$ maximum and minimum timing against ligand concentration) where the ligand is an agonist for both pathways ($\zeta_{+}^{1}=1000,$$\zeta_{-}^{1}=1,$$\zeta_{+}^{2}=100,$$\zeta_{-}^{2}=1,$ top row) and agonist for pathway 1 but an inverse agonist for pathway 2 ($\zeta_{+}^{1}=1000,$$\zeta_{-}^{1}=1,$$\zeta_{+}^{2}=0.01,$$\zeta_{-}^{2}=1,$ bottom row). Constitutive activity for pathway 2 is low ($k_{act-}^{2}=1000,$ left column), medium ($k_{act-}^{2}=10,$ middle column), and high ($k_{act-}^{2}=1,$ left column). Here, $\zeta_{+}^{2}=100$.

Further demonstration of the dynamic and concentration-dependent features of the system is given in Fig. 10, where we observe decreasing peak timing for both pathways where the ligand is an agonist for both, but an increasing trough time at the pathway for which the ligand is an inverse agonist.

#### 3.2.2 Effect of total receptor number on concentration-response

**Fig. 11:**
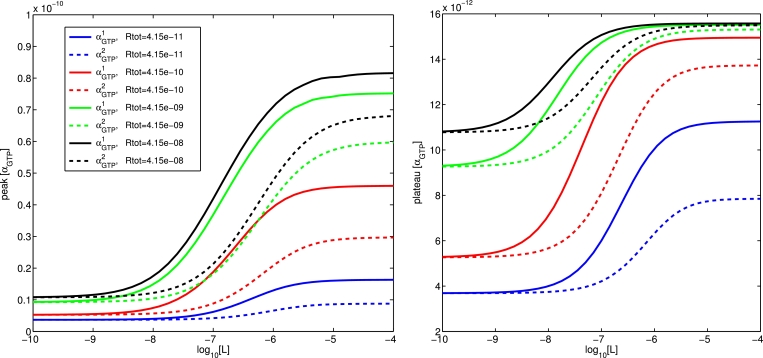
Concentration-response curves for peak and plateau *α_GTP_* for two pathways being agonised by *L*_1_, a ligand with different efficacies for the two pathways ($\zeta_{+}^{1}=1000$ and $\zeta_{+}^{2}=100$), under varying receptor concentrations.

The total concentration of receptor can considerably affect the appearance of bias in a system (Rajagopal et al., 2011). In Fig. 11, we investigate the effect of differing receptor expression by examining concentration-response curves for two pathways being agonised by a ligand (*L*_1_) with different efficacies ($\zeta_{+}^{1}=1000$ and $\zeta_{+}^{2}=100)$ at a range of receptor concentrations *R_tot_* (from 4.15 $\times 10^{-11}$ M to $4.15\times 10^{-8}$ M). As *R_tot_* is decreased, we observe both a rightward shift of the curves (increased *EC*_50_), together with a drop in the maximal responses, for both the peak and plateau values of *α_GTP_*.

**Fig. 12:**
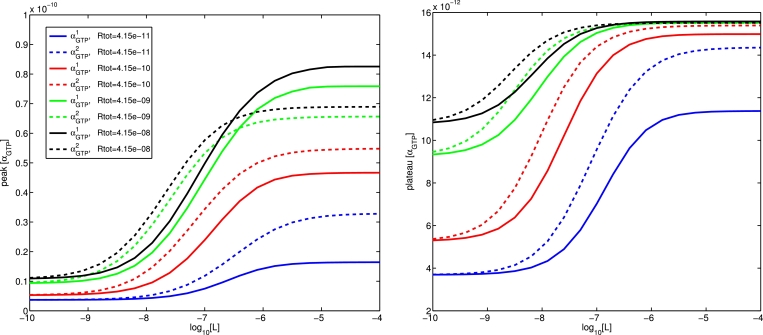
Concentration-response curves for two pathways being agonised by *L*_2_, a ligand with parameters $\zeta_{+}^{1}=2000,$$\zeta_{+}^{2}=100,$$\nu_{+}^{1}=1,$$\nu_{+}^{2}=25,$ vary under changing receptor concentrations.

Whilst overall efficacy depends partly on the preference of the ligand for an active rather than inactive receptor (controlled through variation of the *ζ* parameters), it is important to note that it can also depend on the preference of a ligand-bound receptor for each of the G proteins (mediated by the *ν* parameters). In the case of a system in which the ligand-dependent parameters affecting efficacy are chosen so as to counteract each other ($\zeta_{+}^{1}=2000,$ $\zeta_{+}^{2}=100,$ $\nu_{+}^{1}=1,$ $\nu_{+}^{2}=25$), we see that (Fig. 12) not only the magnitude of the preference for one pathway, but even the direction (in terms of which pathway experiences the higher response) can be affected by a changing receptor concentration. In this case, peak *α_GTP_* exhibits a change in direction, while plateau level does not.

### 3.3 Response surfaces

Parameter sensitivity and concentration-response relations may be conveniently summarised using response surfaces which show the effects of varying two system parameters (Bornheimer, Maurya, Farquhar, Subramaniam, 2004, Bridge, King, Hill, Owen, 2010, Woodroffe, Bridge, King, Hill, 2009). We now use this method to show the sensitivity of simulated response ($\alpha_{GTP}^{1,2}$ peak and plateau) to variations in system parameters, in particular the microaffinity coefficients *ζ, ν* and *μ*.

#### 3.3.1 Effect of *ζ* - the possibility of biphasic relationships

**Fig. 13:**
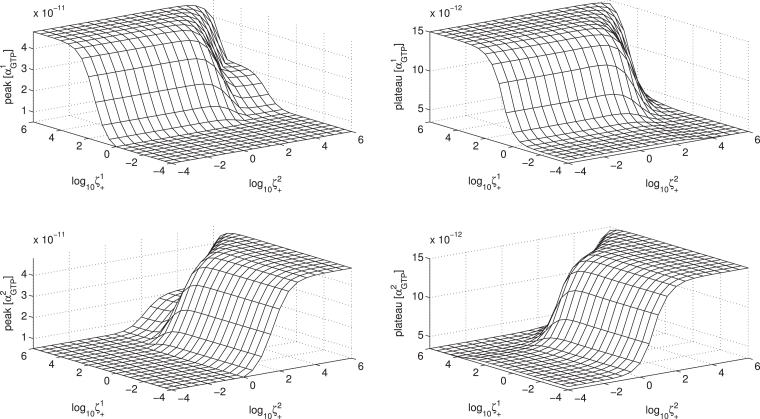
Response surfaces for varying ligand efficacy, for fixed ligand concentration $\left[L\right]=10^{-5}$ M. Parameters $\zeta_{+}^{1}$ and $\zeta_{+}^{2}$ are varied through the spectrum of efficacy for each receptor active state.

In Fig. 13, we see the effect of varying $\zeta_{+}^{1}$ and $\zeta_{+}^{2}$ for a fixed ligand concentration. The reciprocal effects on the two G protein pathways mediated by the competing receptor states are clear. As $\zeta_{+}^{1}$ is increased, both peak and plateau $\alpha_{GTP}^{1}$ increase, accompanied by decreases in $\alpha_{GTP}^{2}$. Furthermore, it is clear that biphasic relationships are possible.

#### 3.3.2 Effect of G protein non-specificity and receptor “cross-states”

**Fig. 14:**
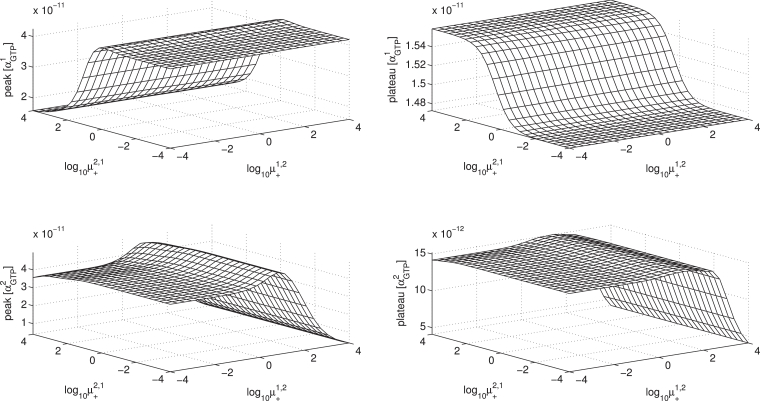
Response surfaces for varying ligand accessibility of receptor cross states for fixed ligand concentration $\left[L\right]=10^{-5}$ M.

Thus far in our computations, we have focussed on systems in which the two G proteins are each specific to a particular active receptor conformation. By setting $\mu^{1,1}=1,\mu^{2,2}=1,\mu^{1,2}=0,\mu^{2,1}=0,$ we have simulated systems whereby G protein 1 can neither activate a pre-coupled receptor towards R^*2^, nor bind to R^*2^, and vice-versa. It is a novel aspect that our model allows receptor “cross-states”, where there is not exclusive specificity of each G protein for one particular active receptor state. We see in Fig. 14 the effects of non-exclusive specificity and accessibility of these cross states on the *α_GTP_* responses. The general trend is that with all cross states signalling (with $k_{GTP}^{j,\theta }=1,\forall \theta$), increasing *μ*_21_ gives a decreased peak $\alpha_{GTP}^{1}$ and slight increase in plateau $\alpha_{GTP}^{1}$.

Our model allows for variation in specificity of not only the G proteins for each receptor conformation, but also the propensity for G protein cycling with respect to these active states, controlled by $k_{GTP+}^{j,\theta }$. With cross states which do not signal (with $k_{GTP}^{j,\theta }=0\;\text{for}\;j\neq \theta$), increasing *μ*_12_ now gives a decreased peak and plateau $\alpha_{GTP}^{2}$ as the general trend, with non-monotonicity, which may be explained by considering the effects on basal conditions. Further explanation and discussion of these effects is given in Appendix B.

#### 3.3.3 Effect of *ν*, the preference of ligand for specific G protein-coupled receptor

**Fig. C1:**
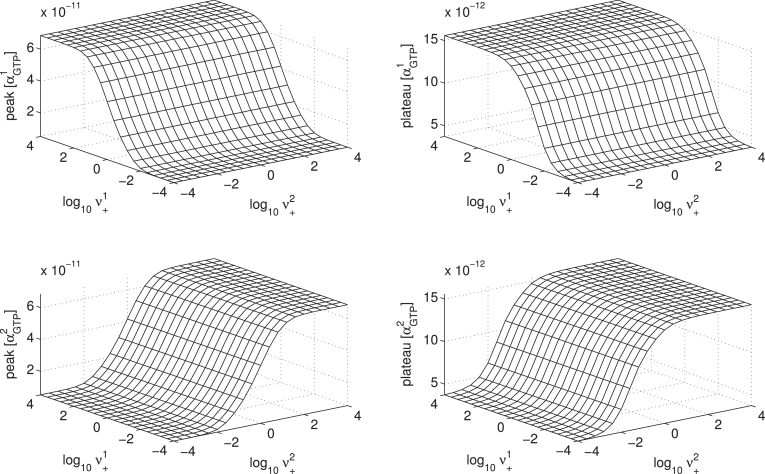
Response surfaces for varying $\nu_{+}^{1,2},$ with expected monotonic relationships. Here, $\left[L\right]=10^{-5}$ M.

The microaffinity constant *ν^θ^* controls the preference of ligand for *RG^θ^* over *R*. The effect of varying *ν^θ^* is as expected, in that increasing *ν^θ^* increases both peak and plateau $\alpha_{GTP}^{\theta }$ (see Fig. C.1 in Appendix C).

## 4 Detecting and quantifying bias

A *balanced agonist* is one which signals with equal efficacy to available downstream pathways, whereas a *biased agonist* has different efficacies for signalling to different pathways (Rajagopal et al., 2011). There is a need to detect and quantify the level of bias towards one pathway over another, considering that physiologically and clinically, certain pathways represent therapeutic targets while others are “side effect” pathways (Gundry et al., 2017). Here, we employ current quantification methods for the level of ligand bias within our two-pathway system.

### 4.1 Bias factors and the operational model of agonism

The operational model of agonism (Black and Leff, 1983) provides a standardised and widely adopted method for estimating ligand affinity and “operational efficacy” parameters from functional response data in the form of hyperbolic concentration-response curves. Briefly, for a single downstream readout *E* resulting from ligand concentration [*A*] at a receptor,

(7) $$E\left(\left[A\right]\right)=E_{max}\frac{\tau [A]}{K_{D}+\left(\tau +1\right)\left[A\right]},$$

where *E_max_* is the maximum response of the system, *K_D_* is the ligand’s equilibrium dissociation constant, and *τ* is a measure of ligand efficacy, in particular measuring the propensity of the ligand *and the system* to yield a response. A modified form of the model is sometimes used to account for nonzero basal responses (Shonberg et al., 2014), namely

(8) $$E\left(\left[A\right]\right)=\text{basal}+\left(E_{max}-\text{basal}\right)\frac{\tau [A]}{K_{D}+\left(\tau +1\right)\left[A\right]}.$$

Further generalisation of this model is possible by introducing a Hill coefficient to the signal transduction sub-model (Black, Leff, 1983, Shonberg, Lopez, Scammells, Christopoulos, Capuano, Lane, 2014). Recently, the operational model has been used to quantify the level of bias in systems exhibiting multi-dimensional efficacy (ie. the activation of multiple pathways at a single receptor). Bias is typically defined with respect to a reference ligand, and *bias factors* are computed using fitted values of *τ* (Rajagopal et al., 2011) or both *τ* and *K_D_* (Gundry, Glenn, Alagesan, Rajagopal, 2017, Kenakin, 2014, Kenakin, Christopoulos, 2013). Here, we follow the transduction coefficients method (Kenakin and Christopoulos, 2013) by defining a transduction coefficient for a ligand *A* at a given pathway as

(9) $$T_{A}=\log_{10}{\left(\frac{\tau }{K_{D}}\right)}_{\text{lig}A}.$$

The difference in transduction coefficients for two ligands *A* and *B* is usually written in Δlog  notation, with

(10) $$\begin{array}{cc} \Delta \log_{10}{\left(\frac{\tau }{K_{D}}\right)}_{\text{lig}A-\text{lig}B} & =\Delta T_{A-B}=T_{A}-T_{B}\\ & =\log_{10}{\left(\frac{\tau }{K_{D}}\right)}_{\text{lig}A}-\log_{10}{\left(\frac{\tau }{K_{D}}\right)}_{\text{lig}B}\\ & =\log_{10}\left({\frac{\tau }{K_{D}}|}_{A}{\frac{K_{D}}{\tau }|}_{B}\right). \end{array}$$

The *relative bias factor* for a ligand *A*, relative to ligand *B*, for pathway 1 over pathway 2, is usually defined by first calculating its logarithm, written in *ΔΔ*log  notation as

(11) $$\begin{array}{cc} \log_{10}{\text{bias}}_{A-B}^{1-2} & =\Delta \Delta \log_{10}{\left(\frac{\tau }{K_{D}}\right)}_{\text{lig}A-\text{lig}B}^{\text{path}1-\text{path}2}=\Delta T_{A-B}^{\text{path}1}-\Delta T_{A-B}^{\text{path}2}\\ & =\log_{10}\left({\frac{\tau }{K_{D}}|}_{A}^{\text{path}1}{\frac{K_{D}}{\tau }|}_{B}^{\text{path}1}{\frac{K_{D}}{\tau }|}_{A}^{\text{path}2}{\frac{\tau }{K_{D}}|}_{B}^{\text{path}2}\right), \end{array}$$

so that

(12) $${\text{bias}}_{A-B}^{1-2}={\frac{\tau }{K_{D}}|}_{A}^{\text{path}1}{\frac{K_{D}}{\tau }|}_{B}^{\text{path}1}{\frac{K_{D}}{\tau }|}_{A}^{\text{path}2}{\frac{\tau }{K_{D}}|}_{B}^{\text{path}2}.$$

### 4.2 Bias factor’s dependence on *ζ* and *ν*

**Fig. 15:**
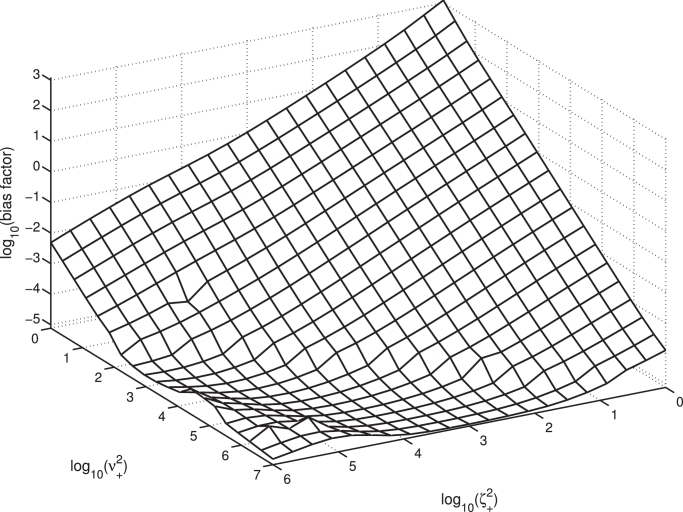
Bias factor surface for fixed ligand 1 parameters, varying ligand 2 parameters *ζ*^2^ and *ν*^2^. Bias factor ${\text{bias}}_{A}^{1-2}$ represents the bias for pathway 1 over pathway 2 signalling. Here, $k_{L+}=10^{5},$$k_{act+}^{1}=k_{act+}^{2}=0.05,$ and $k_{hyd-}^{1}=k_{hyd-}^{2}=10^{-8}$.

The bias factor, ${\text{bias}}_{A}^{1-2}={\frac{\tau }{K_{D}}|}_{A}^{\text{path}1}{\frac{K_{D}}{\tau }|}_{A}^{\text{path}2},$ is a standard measure of a ligand’s bias for effecting a response in pathway 1 over pathway 2. While this is defined in terms of the parameters *τ* and *K_D_* which are fitted to the semi-mechanistic operational model, rather than explicitly in terms of the parameters in our new *α_GTP_* model, we expect correlations between the bias factor and ligand-specific parameters in our model. In particular, when *α_GTP_* is measured at equilibrium and taken as the response *E*, we expect, on the whole, bias factor should increase with decreased *ζ*^2^ and *ν*^2^, which control a ligand’s effect on R^*2^ activation and *G*^2^ coupling to the receptor. In Fig. 15, we show the bias factor ${\text{bias}}_{A}^{1-2}$ for a bank of ligands generated by varying $\zeta_{+}^{2}$ and $\nu_{+}^{2},$ while keeping all other parameters fixed. The correlation is clear. The overall trend is the expected increase in ${\text{bias}}_{A}^{1-2}$ with decreasing $\zeta_{+}^{2}$ and $\nu_{+}^{2},$ while the relationship is approximately a power law over much of the parameter space shown.

### 4.3 Kinetic context and dynamic bias factors

**Fig. 16:**
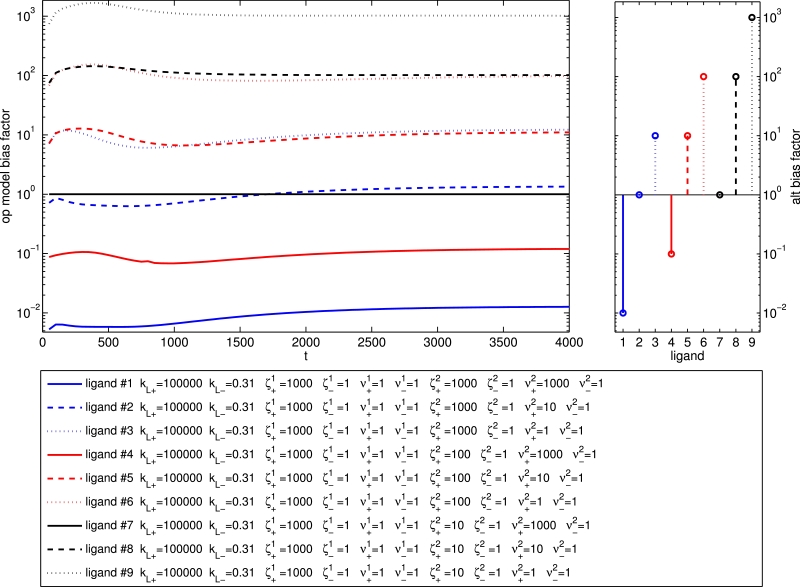
Bias factor dynamics for a bank of nine ligands. The operational model bias factor ${\text{bias}}_{A}^{1-2}$ represents the bias for pathway 1 over pathway 2 signalling is shown for each time point, and the alternative bias factor is shown in the right hand panel. Reference ligand is ligand 7, a strong agonist for pathway 1. Here, $k_{L+}=10^{5},$$k_{act+}^{1}=k_{act+}^{2}=0.05,$$k_{hyd-}^{1}=k_{hyd-}^{2}=10^{-8}$ and $k_{RA-}^{1}=k_{RA-}^{2}=1.3\times 10^{-4}$.

**Fig. 17:**
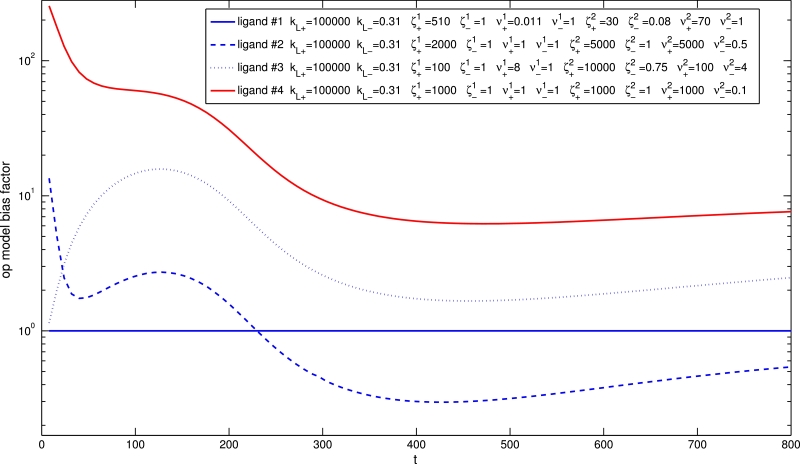
Bias factor dynamics for a bank of four ligands. Bias factor ${\text{bias}}_{A}^{1-2}$ represents the bias for pathway 1 over pathway 2 signalling. Here, $k_{L+}=10^{5},$$k_{act+}^{1}=k_{act+}^{2}=0.01,$$k_{hyd-}^{1}=k_{hyd-}^{2}=10^{-8}$ and $k_{RA-}^{1}=k_{RA-}^{2}=1.3\times 10^{-4}$.

It has recently been demonstrated that binding, activation and signalling dynamics may significantly affect bias measurements, and hence the classification of biased ligands, and that “kinetic context” is an important consideration in the quantification of bias (Herenbrink et al., 2016). Although bias calculations based on the operational model implicitly assume equilibrium conditions, this method is shown to be an effective and simple heuristic approach to investigating and quantifying dynamic bias in Herenbrink et al. (2016). In Fig. 16, we show bias factor time courses for a bank of ligands, generated by constructing a concentration-response curve for each ligand at each time point, then fitting each of these curves to the operational model (using optimisation routines in MATLAB). Here, the bias factor is calculated with respect to the reference ligand (ligand 7), and we see that the long-time bias factor ${\text{bias}}_{A-ref}^{1-2}$ indeed increases with decreased $\zeta_{+}^{2}$ and/or $\nu_{+}^{2}$. We also plot, in the right hand panel, an alternative bias factor based on our model parameters, specifically

(13) $${\text{alt-bias}}_{A-ref}^{1-2}=\frac{\frac{k_{L+,A}}{k_{L-,A}}\left(\frac{\nu_{A}^{1}\zeta_{A}^{1}}{\nu_{A}^{2}\zeta_{A}^{2}}\right)}{\frac{k_{L+,ref}}{k_{L-,ref}}\left(\frac{\nu_{ref}^{1}\zeta_{ref}^{1}}{\nu_{ref}^{2}\zeta_{ref}^{2}}\right)},$$

which should also indicate ligand bias. We note the excellent agreement between the dynamic bias factors from operational model fitting and our alternative bias factor. Dynamically, there is the possibility of a change of order of bias factors, and this phenomenon is even more marked for the bank of ligands shown in Fig. 17. Clearly, the order of bias factors may change dynamically, so the classification of ligands requires consideration of kinetic context, as described in Herenbrink et al. (2016).

### 4.4 Bias dynamics beyond the operational model of agonism

The operational model of agonism is a semi-empirical model used to provide summary measures of signalling efficacy, by implicitly assuming equilibrium conditions; in itself, it cannot be used to simulate biased signalling dynamics. Analysis of biased signalling using a dynamic model which does not rely on the operational model is therefore desirable (for example for fitting to time course data). Our new mechanistic model clearly fulfills this requirement, and will be further applied (beyond our scope here) to define new dynamic bias factors which do not use the operational model assumptions at all.

## 5 Fitting to a model of downstream functional antagonism via biased signalling

Our model outputs thus far have been the *α_GTP_* levels of the two G proteins in the system, which represent responses downstream of ligand binding, and may correspond to a downstream functional experimental readout. We now consider whether our model can be used to explain, and fit to, experimental end point data in a system where biased agonism is suspected.

### 5.1 Experimental method

The adenosine A_1_ receptor (A_1_R) is well known for mediating the protective effects of adenosine in the heart (Donato, Gelpi, 2003, Minamino, 2012). How these effects are brought about is not fully understood, as the A_1_R is able to couple to multiple signalling pathways (Baltos et al., 2016). This makes interpretation of physiological effects difficult to attribute to an individual, signalling pathway. While the A_1_R is a predominantly *G_i_*-coupled receptor, which inhibits the accumulation of the second messenger, cyclic adenosine monophosphate (cAMP), it has been observed that at higher agonist concentrations, the levels of cAMP begin to rise again producing a non-monotonic response profile. This accumulation of cAMP arises through the ability of the A_1_R to switch its G protein coupling and now promote activation of *G_s_* (Baker, Hill, 2007, Cordeaux, IJzerman, Hill, 2004). The extent to which an individual agonist either inhibits or stimulates cAMP production at the A_1_R may vary.

To obtain data to enable fitting of our models, experiments were performed using Chinese hamster ovary-K1 (CHO-K1) cells stably expressing the A_1_ receptor (these cells do not endogenously express any of the four adenosine receptor subtypes, and therefore provide a null background (Knight et al., 2016)), treated with a range of concentrations of a single agonist each time, and the effect on intracellular concentration of cAMP determined (see Appendix D and Knight, Hemmings, Winfield, Leuenberger, Frattini, Frenguelli, Dowell, Lochner, Ladds, 2016, Weston, Lu, Li, Barkan, Richards, Roberts, Skerry, Poyner, Pardamwar, Reynolds, et al., 2015, Weston, Winfield, Harris, Hodgson, Shah, Dowell, Mobarec, Woodlock, Reynolds, Poyner, et al., 2016 for details). In particular, the experiments were carried out for three different agonists individually, namely5’(N-ethyl carboxamido) adenosine (NECA), and two test compounds which bind the A_1_R, denoted here as Compound 6 (Cmpd6) and Compound 20 (Cmpd20) (Knight et al., 2016). The measured response was the accumulated cAMP concentration in the presence of the phosphodiesterase (PDE) inhibitor rolipram, which blocks cAMP degradation.

For each concentration of all agonists, two experiments were performed. Firstly, intact (wild-type) cells were used, which allow for coupling and activation of both *G_i_* and *G_s_* proteins to the A_1_R, thereby allowing activation of both the *G_i_* pathway which inhibits cAMP production via an increased *α*_*GTP, i*_ signal, and the *G_s_* pathway which stimulates cAMP production via an increased *α*_*GTP, s*_ signal. For these cells, the recorded response is the percentage inhibition of cAMP when compared with cells treated with forskolin (which promotes maximal stimulation of cAMP production Barritt, 1992). The second experimental condition is for cells that have been treated with pertussis toxin (PTX), which both inhibits binding of *G_i_* to its receptor and blocks its signal transduction, thereby locking *α_i_* in its inactive, GDP-bound state (Mangmool and Kurose, 2011). For these cells, the recorded response is the percentage stimulation of cAMP when compared with forskolin-stimulated cells. Time courses of cAMP were not recorded, and the signalling readout in each case is taken at the endpoint of the experiment (t=1800 s).

Further details of the experiments are given in Appendix D.

### 5.2 Experimental results

**Fig. 18:**
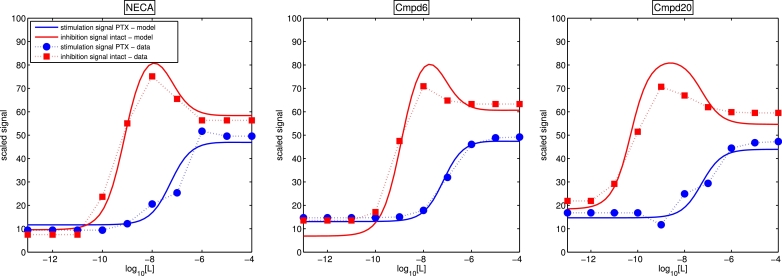
Using the model of biased agonism with functional antagonism at level of *α_GTP_* to fit cAMP readouts (signal_stim_(1800) and signal_inhib_(1800)) for three different ligands (NECA, Cmpd6 and Cmpd20). Experimental data points are given (red squares with dashed lines for percentage cAMP inhibition for wild-type cells, blue circles with dashed lines for percentage cAMP production in PTX-treated cells, each compared with forskolin-treated cells which give maximal cAMP response) for end point readouts over a range of ligand concentrations. Solid curves are the fitted log concentration response curves for our model. (For interpretation of the references to colour in this figure legend, the reader is referred to the web version of this article.)

In Fig. 18, we show the experimental cAMP endpoint signals in response to three ligands individually in turn (NECA, Cmpd6 and Cmpd20) for the two different experimental conditions. For wild-type cells the log concentration response curves *for the inhibition of cAMP* show non-monotonic behaviour with a downturn at higher concentrations, whereas the log concentration response curves *for the production of cAMP* in PTX-treated cells show, with the exception of one data point for the NECA experiment, monotonic behaviour. By blocking the inhibitory pathway, we largely see “standard” monotonic behaviour, which suggests that the non-monotonic wild-type response results from crosstalk between the inhibitory and stimulatory pathways. Since in each case a single ligand has been introduced and A_1_R is the only receptor in the cells, we hypothesise that this target receptor may exhibit biased agonist effects, via two active conformations, one of which is specific to the *G_i_* protein and the other to the *G_s_* protein.

### 5.3 Modelling considerations

Since the data shown in Fig. 18 are hypothesised to result from biased agonism with competition between two activated G protein pathways, we now seek to fit our model to the data, in order to add support to this hypothesis and understand the possible underlying mechanisms.

Within our modelling framework, we let *G*^1^ and *G*^2^ represent the *G_s_* and *G_i_* proteins respectively. We simulate the PTX effect of blocking *G_i_* binding and activation by setting $k_{G+}^{2}=k_{GTP+}^{22}=k_{GRA-}^{2}=0$. Since cAMP is produced in response to *G_s_* activation (Barritt, 1992, Leander, Friedman, 2014), for a simple, minimal model of cAMP levels in PTX-treated cells, with blocked cAMP degradation, we take the cAMP production rate proportional to *α*_*GTP, s*_ levels, so that $\frac{d[cAMP]}{dt}\propto \alpha_{GTP,s},$ and hence the stimulation signal is given by

(14) $${\text{signal}}_{\text{stim}}\left(t\right)=\int_{0}^{t}C_{s}\left[\alpha_{GTP,s}\right]\left(t\right)\;dt\;\;=\;\;\int_{0}^{t}C_{s}\left[\alpha_{GTP}^{1}\left(t\right)\right]\;dt,$$

where *C_s_* is a constant.

For the wild-type cells in which both stimulatory and inhibitory cAMP pathways are intact, we require a model for crosstalk between *G_i_* and *G_s_* pathways. Here we use a simple “functional antagonism” model for the competing effects of these pathways. Functional antagonism refers to the response of a cell in which signalling via one pathway is antagonised by signalling via another pathway, and simple theoretical models have been presented which are based on differences between pathway signals (Leff, Martin, Morse, 1985, Mackay, 1981, Szabadi, 1977). Here, we use such a model where *α*_*GTP, s*_ and *α*_*GTP, i*_ are stimuli to the cAMP stimulatory and inhibitory pathways respectively, and the cAMP production rate is simply a scaled difference between the two *α_GTP_* levels. The inhibition signal is then given by

(15) $$\begin{array}{cc} {\text{signal}}_{\text{inhib}}\left(t\right) & =\int_{0}^{t}C_{i}\left[\alpha_{GTP,i}\right]\left(t\right)-C_{s}\left[\alpha_{GTP,s}\right]\left(t\right)\;dt\\ & =\int_{0}^{t}C_{i}\left[\alpha_{GTP}^{2}\right]\left(t\right)-C_{s}\left[\alpha_{GTP}^{1}\right]\left(t\right)\;dt, \end{array}$$

where *C_i_* is a constant, and *C_s_* is the constant as in (14). Since functionally opposite signalling can result in non-monotonic concentration-response curves with downturns (PliSka, 1994, Szabadi, 1977) such as those seen in the cAMP inhibition curves in Fig. 18, our biased agonism model augmented by the simple functional readout models (14) and (15) may be able to recapitulate the experimental data, at least qualitatively. We proceed to employ parameter estimation methods to pursue a fit to the concentration-response curves for each ligand.

### 5.4 Parameter estimation

We fit the experimental data to the model given by (3a), (3b), (3c), (3d), (3e), (3f), (3g), (3h), (3i), (3j), (3k), (3l), (3m) with $N^{*}=N^{G}=2,$ together with (14) and (15), where simulations are first run to a time of 10^8^ s with [L]=0, to pre-equilibrate the system before ligand addition. For each ligand, the experimental data for the intact and PTX cells were fitted simultaneously, using optimisation algorithms to minimise the squared error between simulation and data points. The methods used were the trust region algorithm implemented in PottersWheel (Maiwald and Timmer, 2008), followed by a genetic algorithm routine implemented in MATLAB (Matlab, 2017). A subset of the kinetic parameters were varied; for each reversible reaction, we fixed one rate constant (typically for the reverse reaction), and allowed one rate constant to float. Further, we consider systems where the active receptor cross states are inaccessible, such that $\mu^{j,\theta }=k_{GTP+}^{j,\theta }=0$ are fixed for *j* ≠ *θ*, since these have been shown to largely have little effect. Fitted parameters for the NECA data set were used as initial parameter guesses for Cmpd6 and Cmpd20, to speed up the overall fitting for these compounds. For each ligand, the model can clearly fit the data very well qualitatively. In Fig. 18, we see that the model fit for the stimulation curve is monotonic, with maximal and basal signals, and *EC*_50_ values in good agreement with the data. Further, the fitted inhibition curve in each case is non-monotonic, with the concentration which gives the peak value in good agreement with the data. The basal, peak and plateau levels are in good agreement with the data, and the model recapitulates the differences in peak “spread” between the three ligands. Values for the fitted parameters are given in Table E.1.

**Fig. 19:**
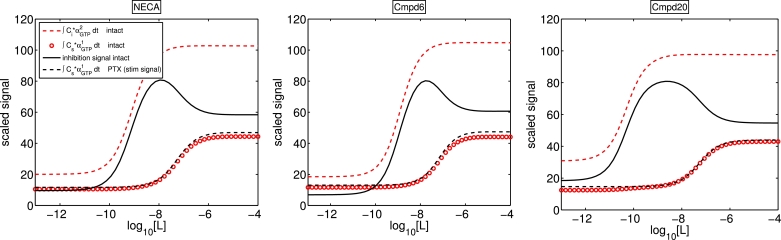
Log concentration response curves for $\int \alpha_{GTP}^{1}\;dt$ and $\int \alpha_{GTP}^{2}\;dt$ levels using fitted parameter values.

In Fig. 19, simulations from the fitted parameter sets for each ligand show the ∫*α_GTP_* contributions to the overall measured signal for both cell types. In each case, the stimulatory responses for the intact cells and the PTX cells are almost indistinguishable, and while the individual ∫*α_GTP_* curves are monotonic, the difference between them for the intact cells is not.

**Fig. 20:**
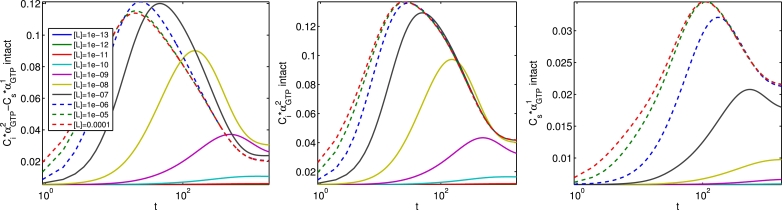
Underlying *α*_*GTP, i*_ and *α*_*GTP, s*_ dynamics for NECA-fitted parameters, for a range of agonist concentrations. Time is on a logarithmic scale to clearly show the peak-plateau time scales.

Having estimated parameters which fit the experimental data (taken at a single time point t=1800 s), we may now simulate the underlying *α_GTP_* dynamics up to this time point. In Fig. 20, we show time courses for *α*_*GTP, i*_ and *α*_*GTP, s*_ levels, using the NECA-fitted parameters. With the ligand being an agonist for both G protein pathways, the peak-plateau *α_GTP_* dynamics are clear, and consistent with the temporal characteristics observed in our earlier numerical simulations. Peak values are monotonic with [*L*], with $\alpha_{GTP}^{1}$ peaking later than $\alpha_{GTP}^{2}$. We conclude that our model recapitulates, and fits to, experimental data well in the cases shown, therefore adding support to the biased agonism conjecture for the experiments discussed, and validating our model. Our functional model for cAMP production is very simple, comprising a linear combination of *α*_*GTP, i*_ and *α*_*GTP, s*_. It is reasonable to expect that a more detailed model of cAMP signalling with more degrees of freedom would result in an even better fit to the data.

## 6 Discussion

Biased agonism is now a widely accepted phenomenon for signalling via GPCRs (Kenakin, Christopoulos, 2013, Urban, Clarke, Von Zastrow, Nichols, Kobilka, Weinstein, Javitch, Roth, Christopoulos, Sexton, et al., 2007), and exploiting this is a potential route to developing novel therapeutics (Kenakin, 2015, Kenakin, Christopoulos, 2013). Theoretical (mathematical) models are key tools towards understanding biased signalling, and have previously been presented for equilibrium conditions (Leff, Scaramellini, Law, McKechnie, 1997, Scaramellini, Leff, 2002). These models have enabled a foundation biased agonism theory to be established, largely at the level of receptor activation. For functional readouts downstream of the receptor, further detail has been added at the level of G protein binding  (Ehlert, 2008), and simple empirical models for pathway signalling (Roche et al., 2013). In this paper, we have developed a new model for biased agonism which includes the detail of G protein activation via a cubic ternary complex/G protein cycle model, with *α_GTP_* as a readout, and serving as an indicator/proxy of pathway activity. This model is general in terms of the number of active receptor conformations and G proteins, and also that it is not specific to any particular pathway; it can thus be used to model biased signalling at any GPCR, and detailed further signalling components can be added downstream of *α_GTP_* to model particular pathways as desired. Potentially, our model could provide a foundation for simulating single-ligand multi-pathway dynamics, such as recent experimental work revealing dynamic biased signalling behaviour at the dopamine D_2_ receptor (Herenbrink et al., 2016).

An important advance in the present study is the analysis of signalling dynamics as predicted by our model. The role of kinetic context in the investigation of biased agonism has recently been highlighted (Herenbrink et al., 2016) and, as such, a model and method for analysing dynamics represents a timely contribution to the literature. A number of dynamic features have been observed here, including the apparent inverse agonist effect of an “antagonist”, dynamic inter-conversion of agonist effect, and the time-dependence of bias factor order. Non-monotonic concentration-response relationships for endpoint signals are possible from our model, for both a single *α_GTP_* readout within a two-pathway system, and downstream crosstalk between two *α_GTP_* signals.

The current standard method for quantifying bias from experimental data uses parameters fitted to the equilibrium operational model of agonism (Black, Leff, 1983, Kenakin, Watson, Muniz-Medina, Christopoulos, Novick, 2012). Calculating bias factors using this empirical model applied to timecourse data shows the dynamic nature of bias (Herenbrink et al., 2016), and our model and computations have reproduced this phenomenon. We propose that such analysis may provide important new insights into, and quantitative characterisation of, experimental timecourse results. The use of the operational model appears to be the current state-of-the-art in bias quantification, but it has a number of limitations: It is empirical rather than mechanistic, it does not consider dynamics, and it does not account for constitutive activity (Stott et al., 2015). An alternative model which includes constitutive activity is given in Slack and Hall (2012), but this equilibrium model has yet to be fully explored with respect to biased signalling. While beyond the scope of our current work, a valuable future investigation will focus on further formulation and definition of dynamic bias factors, including constitutive activity.

We have shown our model to be capable of reproducing endpoint trends in experimental data for cAMP levels in response to ligands at the A_1_R receptor, through multi-pathway (*α*_*GTP, s*_ and *α*_*GTP, i*_) signalling with functionally opposite downstream signals. This endpoint analysis has resulted in parameterisations of the model which then predict the underlying *α_GTP_* dynamics, qualitatively consistent with our earlier agonist-induced simulations. This validation of our model allows us to propose its use for further study of downstream signalling, and fitting to time-course data when it becomes available. For example, for any future dynamic cAMP experimental readouts, our simple functional models (14) and (15) can be used to fit to time-courses, with better fits expected by letting a greater number of parameters float, or using a more detailed cAMP model (eg. Leander and Friedman, 2014). The simulation and fitting in the current work also clearly shows that single-ligand multi-pathway activation at a single receptor provides a mechanism for non-monotonic concentration-response relations either for *α_GTP_* itself or for downstream signals, by way of functional antagonism. While functional signalling experiments often results in monotonic concentration-response curves, relationships with downturns at high concentrations are not uncommon (Calabrese, Baldwin, 2001, PliSka, 1994, Zhu, Liu, Qin, Chen, Liu, 2013), and the current work provides a plausible mechanistic model for understanding such results in systems where multi-pathway signalling via a single receptor is possible.

The mathematical work here represents a theoretical framework for further study of the potential benefits of developing biased agonists as therapeutics. The multidimensionality of GPCR signalling now constitutes a new paradigm in drug discovery, and the potential benefits of new understanding of multi-pathway signalling lie in the development of “functionally selective” drugs which preserve efficacy in target pathways, while minimising activation of unwanted side-effect pathways at the same receptor (Rankovic et al., 2016). Further mechanistic modelling encompassing G protein binding and activation, downstream signalling, dynamics and complexity of the level we have studied here is acknowledged as a potentially very valuable advance towards such drug discovery goals (Stott, Hall, Holliday, 2015, Urban, Clarke, Von Zastrow, Nichols, Kobilka, Weinstein, Javitch, Roth, Christopoulos, Sexton, et al., 2007).

## Author contributions

LJB developed models, performed computations and analysis, and wrote the manuscript. JM performed computations and analysis, and wrote the manuscript. EF performed wet-lab experiments. IW performed wet-lab experiments. GL developed models, analysed data and wrote the manuscript.

## Figures and Tables

**Table A1:** Parameter values for 2 G protein, 2 active receptor state model.

Label	Meaning	Cell or ligand specific	Value	Units	Source
$k_{L+}$	Ligand binding rate	Ligand	9.40E+04	M${}^{-1}$ s${}^{-1}$	Bridge et al. (2010)
$k_{L-}$	Ligand unbinding rate	Ligand	3.10E-01	s${}^{-1}$	”
$k_{act+}^{1}$	Receptor activation rate to R^*1^	Cell	1.00E+00	s${}^{-1}$	”
$k_{act-}^{1}$	Receptor deactivation rate from R^*1^	Cell	1.00E+03	s${}^{-1}$	”
$k_{act+}^{2}$	Receptor activation rate to R^*2^	Cell	1.00E+00	s${}^{-1}$	”
$k_{act-}^{2}$	Receptor deactivation rate from R^*2^	Cell	1.00E+03	s${}^{-1}$	”
$k_{G+}^{1}$	G protein 1 binding rate	Cell	1.00E+08	M${}^{-1}$ s${}^{-1}$	”
$k_{G-}^{1}$	G protein 1 unbinding rate	Cell	1.00E-01	s${}^{-1}$	”
$k_{G+}^{2}$	G protein 2 binding rate	Cell	1.00E+08	M${}^{-1}$ s${}^{-1}$	”
$k_{G-}^{2}$	G protein 2 unbinding rate	Cell	1.00E-01	s${}^{-1}$	”
$k_{GRA+}^{1}$	G protein 1 reassociation rate	Cell	7.00E+05	M${}^{-1}$ s${}^{-1}$	”
$k_{GRA-}^{1}$	G protein 1 dissociation rate	Cell	1.30E-03	s${}^{-1}$	”
$k_{GRA+}^{2}$	G protein 2 reassociation rate	Cell	7.00E+05	M${}^{-1}$ s${}^{-1}$	”
$k_{GRA-}^{2}$	G protein 2 dissociation rate	Cell	1.30E-03	s${}^{-1}$	”
$k_{hyd+}^{1}$	Hydrolysis rate of $G\alpha_{GTP}^{1}$	Cell	1.00E-02	s${}^{-1}$	”
$k_{hyd-}^{1}$	Exchange rate of GTP for GDP at $G_{\alpha }^{1}$	Cell	1.00E-04	s${}^{-1}$	”
$k_{hyd+}^{2}$	Hydrolysis rate of $G\alpha_{GTP}^{2}$	Cell	1.00E-02	s${}^{-1}$	”
$k_{hyd-}^{2}$	Exchange rate of GTP for GDP at $G_{\alpha }^{2}$	Cell	1.00E-04	s${}^{-1}$	”
$k_{GTP+}^{1,1}$	R^*1^*G*^1^ dissociation rate	Cell	1.00E+00	s${}^{-1}$	”
$k_{GTP+}^{1,2}$	R^*1^*G*^2^ dissociation rate	Cell	1.00E+00	s${}^{-1}$	”
$k_{GTP+}^{2,1}$	R^*2^*G*^1^ dissociation rate	Cell	1.00E+00	s${}^{-1}$	”
$k_{GTP+}^{2,2}$	R^*2^*G*^2^ dissociation rate	Cell	1.00E+00	s${}^{-1}$	”
$\nu_{+}^{\theta }$	Forward cooperativity factor for ligand binding a *G^θ^* bound receptor	Ligand	1.00E+00		”
$\nu_{-}^{\theta }$	Backward cooperativity factor for ligand binding a *G^θ^* bound receptor	Ligand	1.00E+00		”
$\zeta_{+}^{j}$	Forward cooperativity factor for ligand-bound *R^j^* activation	Ligand	1.00E+03		”
$\zeta_{-}^{j}$	Backward cooperativity factor for ligand-bound *R^j^* activation	Ligand	1.00E+00		”
$\mu_{+}^{j,\theta }$	Forward cooperativity factor for *G^θ^*-bound *R^j^* activation	Cell	1.00E+00 (j=θ), 0 (*j* ≠ *θ*)		”
$\mu_{-}^{j,\theta }$	Backward cooperativity factor for *G^θ^*-bound *R^j^* activation	Cell	1.00E+00		”
*R_tot_*	Total receptor concentration	Cell	4.15E-10	M	”
$G_{tot}^{1}$	Total *G*^1^ concentration	Cell	4.15E-10	M	”
$G_{tot}^{2}$	Total *G*^2^ concentration	Cell	4.15E-10	M	”
*L_tot_*	Total Ligand concentration	Ligand	1.00E-07	M	”

**Fig. B1:**
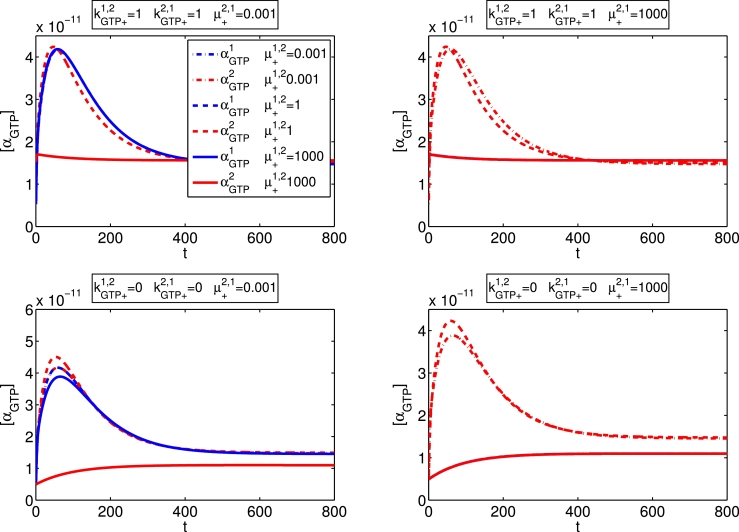
Time courses for varying agonist accessibility of receptor cross states. Here, $\left[L\right]=10^{-5}$ M.

**Table E1:** Parameter estimates for fitting biased agonism with functional antagonism model to cAMP data (see Fig. 18).

Parameter	Cmpd6	Cmpd20	NECA
$k_{L+}$	1.20E+04	7.49E+03	1.52E+04
$\zeta_{+}^{1}$	4.47E+03	1.52E+02	4.55E+03
$\zeta_{+}^{2}$	2.70E+04	7.00E+03	2.54E+04
$\nu_{+}^{1}$	5.82E-01	1.22E+01	5.82E-01
$\nu_{+}^{2}$	6.00E+00	2.54E+02	8.36E+00
$k_{act+}^{1}$	8.80E-01	1.44E+00	9.32E-01
$k_{act+}^{2}$	6.47E-01	1.40E+00	5.94E-01
$k_{G+}^{1}$	6.34E+08	5.69E+08	6.55E+08
$k_{G+}^{2}$	2.89E+09	3.72E+09	2.36E+09
$k_{GRA+}^{1}$	7.55E+05	7.99E+05	7.63E+05
$k_{GRA+}^{2}$	4.10E+05	3.50E+05	3.28E+05
$k_{hyd+}^{1}$	2.42E-03	4.40E-03	2.42E-03
$k_{hyd+}^{2}$	3.20E-03	3.00E-03	3.30E-03
$k_{GTP+}^{1,1}$	1.90E-01	2.01E-01	1.75E-01
$k_{GTP+}^{2,2}$	1.87E-01	2.27E-01	1.87E-01
$\mu_{+}^{1,1}$	1.28E+00	2.16E+00	1.28E+00
$\mu_{+}^{2,2}$	8.60E-01	8.50E-01	8.60E-01
*R_tot_*	4.15E-10	4.15E-10	4.15E-10
$G_{tot}^{1}$	3.79E-10	4.80E-10	4.49E-10
$G_{tot}^{2}$	8.22E-10	7.20E-10	8.28E-10
*C_s_*	4.30E+08	4.36E+08	3.24E+08
*C_i_*	4.40E+08	5.30E+08	4.99E+08

**Fig. F1:**
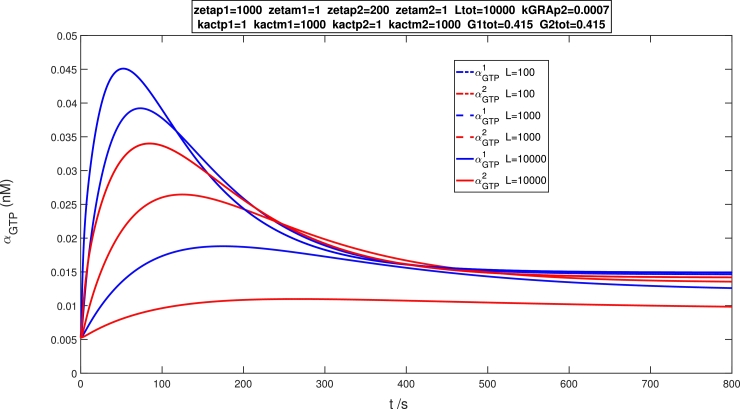
Agonist-agonist time courses, equivalent to Fig. 4, but using new rescaled parameter set. We see that the *α_GTP_* concentrations are simply rescaled.

**Fig. F2:**
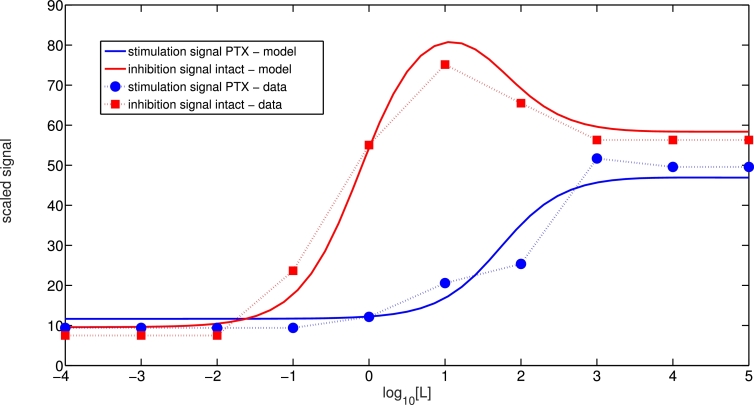
Parameter fitting for NECA experiment, using new rescaled parameter set.

**Table F1:** Parameter values for 2 G protein, 2 active receptor state model, now measuring concentrations in nM.

Label	Meaning	Value (typical simulation)	Value (fitted for NECA)	Units
$k_{L+}$	Ligand binding rate	9.40E-05	1.52E-05	nM${}^{-1}$ s${}^{-1}$
$k_{G+}^{1}$	G protein 1 binding rate	1.00E-01	6.55E-01	nM${}^{-1}$ s${}^{-1}$
$k_{G+}^{2}$	G protein 2 binding rate	1.00E-01	2.36E+00	nM${}^{-1}$ s${}^{-1}$
$k_{GRA+}^{1}$	G protein 1 reassociation rate	7.00E-04	7.63E-04	nM${}^{-1}$ s${}^{-1}$
$k_{GRA+}^{2}$	G protein 2 reassociation rate	7.00E-04	3.28E-04	nM${}^{-1}$ s${}^{-1}$
*R_tot_*	Total receptor concentration	4.15E-1	4.15E-01	nM
$G_{tot}^{1}$	Total *G*^1^ concentration	4.15E-1	4.49E-01	nM
$G_{tot}^{2}$	Total *G*^2^ concentration	4.15E-1	8.28E-01	nM
*L_tot_*	Total ligand concentration	1.00E+02	–	nM

## Appendix A Parameter values

In Table A.1, we give a base parameter set for all computations. Any variations from this parameter set are shown in figure titles and captions.

## Appendix B Receptor cross states

In Fig. B.1, we show simulated time courses for *α_GTP_*, under variation of receptor cross state activation and accessibility. In the top row, we allow activation of both G proteins by either active state (by setting $k_{GTP}^{j,\theta }=1\;\forall j,\theta$), and vary the propensity for G protein binding to the active states by varying $\mu_{+}^{1,2}$. With $\mu_{+}^{2,1}=0.001$ (so that there is very little *R*^*2^*G*^1^, top left plot), as $\mu_{+}^{1,2}$ increases, basal $\alpha_{GTP}^{2}$ increases due to increased signalling via pre-coupled *R*^*1^*G*^2^. Also there is a slight increase in plateau $\alpha_{GTP}^{2}$. In this case, the perhaps unexpected trend in the peak response, whereby peak $\alpha_{GTP}^{2}$ does not increase with $\mu_{+}^{1,2},$ is due to the fact that for large $\mu_{+}^{1,2},$ the basal conditions are “near equilibrium”, and the peak may increase or decrease with increased $\mu_{+}^{1,2}$. The $\alpha_{GTP}^{1}$ equilibrium and dynamics are not significantly affected by changes in $\mu_{+}^{1,2}$. Performing the same simulations with $\mu_{+}^{2,1}=1000$ (top right plot), $\alpha_{GTP}^{1}$ dynamics are again largely unaffected by the variation in $\mu_{+}^{1,2},$ but this time the system is closer to equilibrium and $\alpha_{GTP}^{1}$ has a lower peak than for $\mu_{+}^{2,1}=0.001$ .

In the case where cross states are accessible in the G protein binding sense, but there is specificity with respect to g protein activation (so $k_{GTP}^{1,2}=k_{GTP}^{1,2}=0$), for increasing $\mu_{+}^{1,2},$ we now have more of *G*^2^ trapped in a non-signalling complex *R*^*1^*G*^2^, giving reduced basal and equilibrium $\alpha_{GTP}^{2}$. The basal and equilibrium levels are monotonic with $\mu_{+}^{1,2},$ but the peak levels are not. For $\mu_{+}^{2,1}=0.001,$ as $\mu_{+}^{1,2}$ increases, more *R*^*1^ is trapped in non-signalling complexes, so there is less *R*^*1^*G*^1^ available to signal, resulting in a lower $\alpha_{GTP}^{1}$. For $\mu_{+}^{2,1}=1000,$ we see the same trends, but with lower $\alpha_{GTP}^{1,2}$ signals, since there is more non-signalling *R*^*2^*G*^1^.

## Appendix C Effect of *ν*

In Fig. C.1, we show that the effect of varying *ν^θ^* (the microaffinity constant *ν^θ^* that controls the preference of ligand for *RG^θ^* over *R*) is as expected; increasing *ν^θ^* increases both peak and plateau $\alpha_{GTP}^{\theta }$.

## Appendix D Further experimental detail for cAMP experiments

CHO-K1 cells, expressing the A_1_R were routinely grown in Hams F-12 media (supplemented with 10% FBS), at 37 °C, in a humidified atmosphere, containing 5% CO_2_. Where *G_s_* assays were performed, cells were pre-treated, for 16–18 h with 200 ng/ml PTX. Upon day of assay, cells were harvested and brought to single cells suspension using trypsin (containing 0.05 EDTA). Cells were washed and resuspended in stimulation buffer (PBS containing 0.1% BSA and 25 µM rolipram). Cells were seeded onto 384-well, white, optiplates and stimulated with either agonist alone, or co-stimulated with agonist and 10 µM forskolin, for 30 min. cAMP levels were then detected using a LANCE^®^ cAMP detection kit (PerkinElmer, Boston, MA), and plates read using a LB 940 multimode microplate reader (Berthold technologies, Germany) (excitation: 340 nm, emission: 665 nm).

## Appendix E Parameter estimates for cAMP experiments

In Table E.1, we show parameter estimates for the experimental data shown in Fig. 18. Inspection of the values shows that ligand-dependent parameter values vary over orders of magnitude across the three experiments, while cell-only parameter value estimates are all within an order of magnitude of each other, as expected. Also, our estimates for the G protein totals are consistent with the observation that in most membranes, the amount of *G_i_* protein exceeds the amount of *G_s_* (Barritt, 1992).

## Appendix F Parameter estimates, units and scales

We remark here that, in keeping with (Bridge, King, Hill, Owen, 2010, Woodroffe, Bridge, King, Chen, Hill, 2010), we have reported concentrations in units of molar (M), and performed computations using parameters in the units shown in Table A.1. While working in these units requires dimensional parameter values on widely different orders of magnitude, we encounter no numerical difficulties related to roundoff errors or catastrophic cancellation. An alternative choice of concentration units suggested by Table A.1 is nM, so that the receptor and G protein concentrations, if scaled with their initial values, would be O(1). In Table F.1 we show the variable and parameter values affected by this change of units, which gives a smaller range of orders of magnitude in our parameter list. Re-computing with the new concentration units, for both simulation and parameter estimation, gives the same dynamic results and fitting as with the original parameter set (see, eg., Fig. F.1). In Table F.1, we also show sample parameter estimates for the experimental data using NECA as the ligand. Rescaling all concentrations (changing units to nM rather than M), we reduce the range of orders of magnitude of the parameters. Further, we have taken initial parameter estimates from this rescaled set, and perturbed them, then re-run genetic algorithms, and recover this rescaled set as our best fit (see Fig. F.2). This indicates that the original fitting routine did not suffer any numerical difficulties due to the range of orders of magnitude.

Beyond this simple change of units, careful scaling of both the state and time variables by representative values would yield a fully nondimensional system which would allow for identification of relative magnitudes of dimensionless parameters which control the dynamics, as in Woodroffe, Bridge, King, Chen, Hill, 2010, Woodroffe, Bridge, King, Hill, 2009 and Bridge et al. (2010). Such an approach is useful for (smaller) ODE systems for which further analytical work may be tractable, but is beyond our scope here.
